# Current Landscape of Glycogen Synthase Kinase-3β PET Tracers: Progress, Limitations, and Perspectives—A Narrative Review

**DOI:** 10.3390/ph19091457

**Published:** 2026-09-14

**Authors:** Hualong Chen, Lu Zhang, Bingke Jiao, Xinyu Sun, Leyi Guan, Ruoran Niu, Boxin Li, Xue Jiang, Zehui Wu

**Affiliations:** 1 Beijing Institute of Brain Disorders, Beijing Key Laboratory of Innovation and Translation of Central Nervous System Radiopharmaceuticals, Capital Medical University, Beijing 100069, China; chenhl@ccmu.edu.cn (H.C.); 112024040463@mail.ccmu.edu.cn (L.Z.); 122025011618@mail.ccmu.edu.cn (B.J.); sunxinyu@mail.ccmu.edu.cn (X.S.); 2 School of Pharmaceutical Sciences, Capital Medical University, Beijing 100069, China; 2129030@mail.ccmu.edu.cn (L.G.); younr024@mail.ccmu.edu.cn (R.N.); 20060823lbx@mail.ccmu.edu.cn (B.L.); 3 Department of Nuclear Medicine, The Second Affiliated Hospital of Chengdu Medical College, China National Nuclear Corporation 416 Hospital, No. 4, Fourth Section, North Ring Road, Chenghua District, Chengdu 610051, China; 4 Department of Nuclear Medicine, Tongren People’s Hospital, Tongren 554300, China

**Keywords:** glycogen synthase kinase-3β, positron emission tomography, radiotracers, molecular imaging, clinical translation

## Abstract

**Background/Objectives:** Glycogen synthase kinase-3β (GSK-3β) serves as a crucial molecular target for Alzheimer’s disease and other neurological disorders. Positron emission tomography (PET) enables non-invasive quantification of GSK-3β in vivo, offering value for early diagnosis and mechanistic studies. However, despite two decades of effort, no GSK-3β PET tracer has yet entered clinical trials. **Methods:** This review systematically summarizes the radiochemical synthesis and preclinical evaluation of major GSK-3β tracer classes, comparing synthetic accessibility, target affinity, brain uptake, P-gp efflux, metabolic stability, radiometabolite profiles, and blocking experiments. The literature was systematically searched in PubMed and Web of Science databases (2005–2026). Several key obstacles impeding clinical progression of GSK-3β PET radiotracers are identified. **Results:** First, P-gp efflux markedly reduces brain tracer accumulation, rendering even sub-nanomolar high-affinity ligands ineffective for brain imaging. Second, interfering factors such as brain-penetrant radiometabolites of [^11^C]OCM-44 and the extremely low plasma free fraction of [^18^F]OCM-50 greatly impair the quantitative accuracy of tracer uptake. Third, the paradoxical increase in brain uptake of isonicotinamide tracers upon blocking remains an unresolved confounding factor. Moreover, rodent data fail to reliably predict tracer performance in higher species, and to date, no tracer has been directly validated against GSK-3β expression in patient brain tissue. **Conclusions:** This review proposes a tiered validation framework comprising four levels: assessment of P-gp efflux, metabolite-corrected arterial input function, low-affinity enantiomer controls, and human brain autoradiography. The framework provides a rigorous basis for screening candidates and a systematic reference for developing next-generation GSK-3β PET tracers.

## 1. Introduction

Glycogen synthase kinase-3β (GSK-3β) is an intracellular serine/threonine kinase highly enriched in the mammalian central nervous system (CNS) [1,2]. Unlike most kinases, GSK-3β is constitutively active under resting conditions, and its activity is tightly regulated through complex phosphorylation events. Phosphorylation at serine 9 (Ser9) inhibits its activity, which is primarily mediated by upstream signaling pathways such as PI3K/Akt and Wnt, whereas phosphorylation at tyrosine 216 (Tyr216) promotes its activation [3,4,5]. Under physiological conditions, GSK-3β regulates neurogenesis, axonal growth, synaptic plasticity, and memory formation by phosphorylating diverse substrate proteins [6,7,8,9]. Distinguished from cell surface receptors, this intracellular kinase acts on its substrates across multiple subcellular compartments.

**Figure 1:**
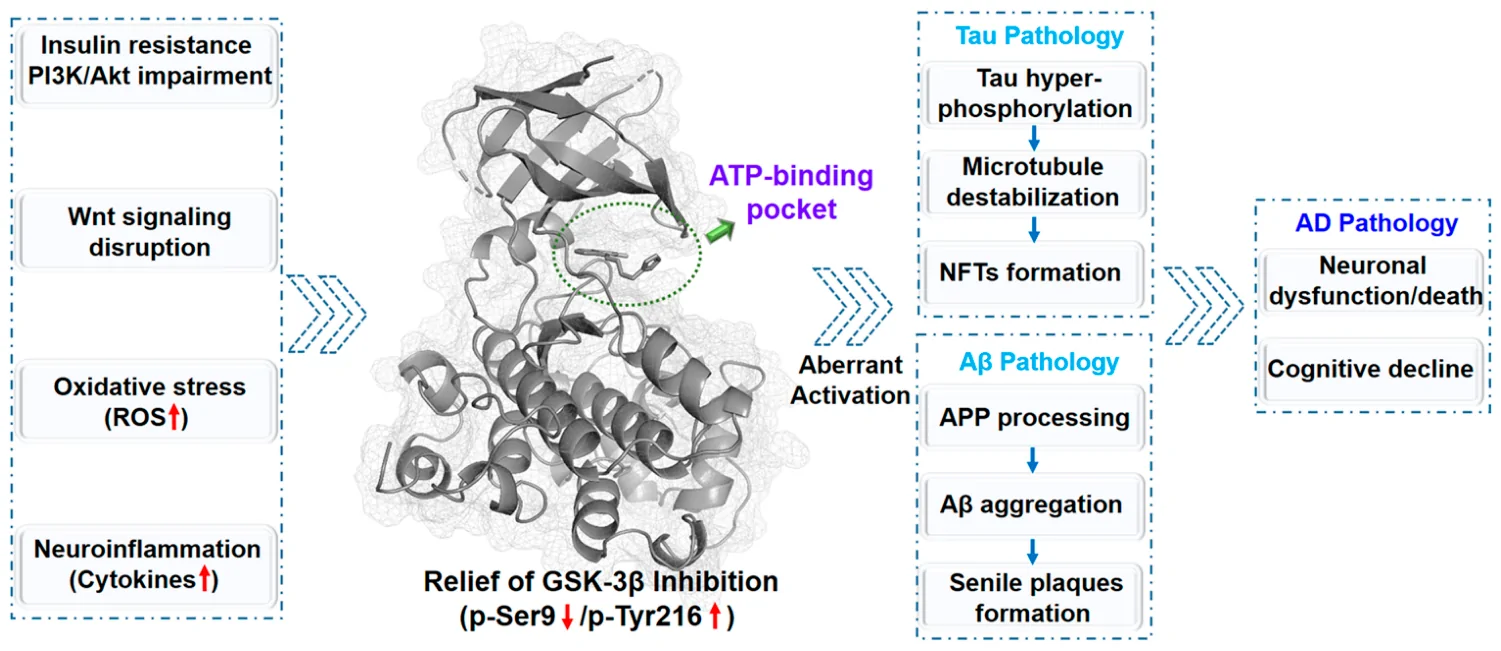
GSK-3β: a key regulator of AD pathology. GSK-3β integrates upstream signals to drive AD pathologies; its inhibition rescues deficits, making it a key target for AD intervention and imaging. Structural representation of GSK-3β (PDB ID: 5K5N [22], from RCSB PDB database) with the ATP-binding pocket highlighted in the structure (rendered with PyMOL [23]). Red arrows: increase/decrease in the corresponding indicator. Green arrow: ATP-binding pocket. Blue arrows: activation/promotion processes.

In Alzheimer’s disease (AD), GSK-3β regulatory pathways are disrupted, resulting in aberrant hyperactivation (Figure 1). Pathological triggers such as insulin resistance-induced PI3K/Akt dysfunction, impaired Wnt signaling, oxidative stress, and neuroinflammation collectively relieve GSK-3β inhibition [10,11]. This excessive activity drives both core pathological hallmarks of AD. As a primary tau kinase, GSK-3β induces tau hyperphosphorylation, which disrupts microtubules and ultimately leads to neurofibrillary tangle (NFT) formation [12,13,14]. It also modulates amyloid precursor protein (APP) processing to promote amyloid-β (Aβ) production and aggregation, facilitating senile plaque deposition [12]. Notably, Aβ oligomers further activate GSK-3β, creating a pathogenic positive feedback loop that accelerates disease progression [15,16] (Figure 1). 

Therapeutic studies have further confirmed GSK-3β as a key mediator of AD pathogenesis. In 3×Tg-AD mice, increased inhibitory phosphorylation at GSK-3β Ser9 (pSer9) reduced tau hyperphosphorylation, suppressed neuronal apoptosis, and improved cognitive performance [17,18]. GSK-3β therefore functions as a central hub in the AD signaling network, integrating multiple upstream pathological stimuli and propagating signals that drive both core AD pathologies. Given this unique role, GSK-3β represents an attractive target for therapeutic development and molecular imaging.

Positron emission tomography (PET) enables in vivo quantitative assessment of target protein expression and activity using specific radiotracers [19,20,21]. Thus, GSK-3β is both a biomarker of AD pathological burden and disease progression, and a key signaling node that integrates upstream pathological signals to drive downstream core pathologies. Non-invasive, in vivo quantitative imaging of cerebral GSK-3β therefore holds significant value.

This narrative review was compiled through systematic searches of PubMed and Web of Science (2005–2026) using combinations of keywords including “GSK-3β,” “PET,” “radiotracer,” “glycogen synthase kinase-3,” “imaging probe,” and related terms. Reference lists of retrieved articles were manually screened. Conference abstracts without full publications were excluded; selected in vitro-only studies with relevant mechanistic insights were included where appropriate.

## 2. Biological Basis of GSK-3β

### 2.1. Structural Features of GSK-3β

The GSK-3 family comprises two highly homologous isoforms, GSK-3α (51 kDa) and GSK-3β (47 kDa), encoded by separate genes. Their catalytic domains share up to 98% sequence identity, with differences confined mainly to the *N-* and *C*-terminus regions. GSK-3β is the predominant isoform in most brain regions, and its expression increases with age [24,25,26]. The substrate-binding domain of GSK-3β shows low conservation among kinases. Its surface hydrophobic groove serves as a key region for substrate recognition, laying a structural foundation for the development of substrate-competitive inhibitors [27]. Multiple druggable pockets are distributed at the surface of GSK-3β, including the ATP-binding site, substrate-binding site, axin/FRAT-binding site, and potential allosteric sites [28,29,30]. Notably, within the ATP-binding pocket, Asp133 of GSK-3β and Glu196 of GSK-3α represent the only non-conserved residues between the two isoforms. This “Asp-Glu switch” provides a structural foundation for the design of isoform-selective inhibitors [31,32].

Based on the above structural features, researchers have synthesized a variety of small-molecule GSK-3β inhibitors and resolved their co-crystal structures bound to this kinase. The co-crystal structure of PF-367 complexed with GSK-3β (PDB: 5K5N) confirms that this compound acts as a typical type I kinase inhibitor occupying the ATP-binding pocket [22]. Co-crystal structures of imidazo [1,2-b]pyridazine-derived inhibitors also offer valuable structural references for drug optimization [33].

In addition, covalent GSK-3β inhibitors have attracted extensive research attention [34,35,36,37]. Tideglusib, an irreversible GSK-3β inhibitor, has entered Phase II clinical trials, yet its inhibitory efficacy is not fully mediated by Cys199 [33]. By contrast, thiazolidine-2-thione derivatives and aza-indolyl maleimide-based inhibitors irreversibly target Cys199 with nanomolar potency [35,36]. Similarly, incorporating a halomethylketone group converts reversible GSK-3 inhibitors into irreversible analogs, also with nanomolar IC_50_ values [37].

### 2.2. GSK-3β and Diseases

Aberrant activation of GSK-3β is closely correlated with a broad range of human diseases, including neurological disorders, psychiatric illnesses, metabolic disorders, cancers, and inflammatory diseases.

#### 2.2.1. Alzheimer’s Disease

AD is the most extensively studied GSK-3β-associated neurodegenerative disorder, with GSK-3β playing a central role in its pathogenesis. In AD brains, it remains excessively activated and contributes to tau and Aβ toxicity, oxidative stress, neuroinflammation, and synaptic dysfunction [38,39,40]. GSK-3β hyperphosphorylates tau at multiple sites, most notably Ser396, Ser199, and Ser404, reducing tau’s microtubule affinity and promoting neurofibrillary tangle formation [41,42,43]. Hyperactivated GSK-3β colocalizes with tangles in post-mortem AD brain tissue [42]. It also accelerates Aβ aggregation, and pharmacological GSK-3β inhibition alleviated Aβ-induced neurotoxicity and cognitive deficits in transgenic AD mice [44]. Excessive GSK-3β activity further promotes neuroinflammation, neuronal injury, and apoptosis. In induced pluripotent stem cell-derived neurons from familial and sporadic AD patients, elevated Aβ_1–40_ levels, increased active GSK-3β that lack Ser9 phosphorylation, and higher phospho-tau/total tau ratios were strongly correlated [45,46].

#### 2.2.2. Parkinson’s Disease

Beyond AD, GSK-3β dysregulation is also tightly linked to the pathogenesis of Parkinson’s disease. Substantial evidence reveals that GSK-3β hyperactivation elevates the expression of α-synuclein, the main component of Lewy bodies, a typical pathological hallmark of PD [47,48]. In PD animal models, GSK-3β inhibition reduced α-synuclein aggregation and neurotoxicity, indicating that GSK-3β is a promising therapeutic target for PD. Additionally, GSK-3β participates in PD-associated neuroinflammation and mitochondrial dysfunction, which further aggravates dopaminergic neuron loss [49,50].

#### 2.2.3. Huntington’s Disease

Similarly, aberrant GSK-3β activity has been implicated in Huntington’s disease (HD) [51,52]. HD is a neurodegenerative disorder caused by Huntingtin (*HTT*) gene mutation, characterized by progressive neuronal loss in the striatum and cerebral cortex. Studies verified increased GSK-3β activity in HD brain tissue, which correlated with mutant Huntingtin aggregation and cytotoxicity. Suppression of GSK-3β reduced neuronal apoptosis and mitochondrial dysfunction that is triggered by mutant Huntingtin protein [53].

#### 2.2.4. Tauopathies

In addition to its role in specific neurodegenerative diseases, GSK-3β-mediated tau hyperphosphorylation serves as a shared pathological mechanism across various tauopathies [54]. Tauopathies are a group of neurodegenerative diseases defined by abnormal tau protein aggregation, including progressive supranuclear palsy (PSP), corticobasal degeneration (CBD), Pick’s disease (PiD), argyrophilic grain disease (AGD), and frontotemporal dementia with parkinsonism linked to chromosome 17 (FTDP-17) [54]. In these diseases, abnormal GSK-3β activation causes tau hyperphosphorylation and subsequent aggregation into neurofibrillary tangles in specific brain regions, ultimately leading to neuronal dysfunction and death [55].

#### 2.2.5. Bipolar Disorder and Depression

GSK-3β dysregulation extends beyond neurodegenerative conditions to bipolar disorder and depression. Disordered GSK-3β signaling pathways underlie the pathogenesis of bipolar disorder (BD) [56]. Lithium, a classic mood stabilizer, exerts therapeutic effects via inhibiting GSK-3β. Its mechanisms include direct competitive binding against magnesium ions and indirect promotion of inhibitory Ser9 phosphorylation. Studies have shown that low-dose lithium regulates GSK-3β activity in a region-specific manner in the brain. Such regional differences may explain the clinical heterogeneity among BD patients [57]. Moreover, disrupted sleep–wake rhythm is a core endophenotype of depression and bipolar disorder. GSK-3β is critical for circadian rhythm and sleep–wake cycle regulation, and its dysfunction contributes to sleep disturbances in these psychiatric disorders [58].

#### 2.2.6. Type 2 Diabetes Mellitus

The pathological roles of GSK-3β are not restricted to the central nervous system. GSK-3β plays a core regulatory role in glucose metabolism. It phosphorylates and inactivates glycogen synthase, thereby inhibiting glycogen synthesis and inducing hyperglycemia [53,59,60,61]. Both expression level and enzymatic activity of GSK-3β are significantly upregulated in type 2 diabetic patients. GSK-3β inhibition mimics insulin effects by boosting glycogen synthesis and relieving insulin resistance, making GSK-3β a valuable therapeutic target for type 2 diabetes [61]. Derivatives of the marine natural product meridianin C act as potent GSK-3β inhibitors with improved oral bioavailability, offering novel candidate drugs for diabetes treatment [60].

#### 2.2.7. Cancer

In addition to its metabolic and neurological roles, GSK-3β also participates in tumorigenesis. GSK-3β facilitates tumor progression in multiple malignancies, such as colorectal cancer, pancreatic cancer, prostate cancer, glioblastoma, and leukemia [62,63,64]. It drives tumor initiation and progression by promoting proliferation, survival, migration, invasion, and chemoresistance, and serves as a cancer cell marker via aberrant nuclear accumulation in certain tumors. Several clinically investigated GSK-3β inhibitors, including Tideglusib [64] and 9-ING-41 [65], are being developed for the treatment of both neurological diseases and cancers.

#### 2.2.8. Inflammatory Diseases

In addition, GSK-3β functions as a critical regulator of immune and inflammatory responses, acting via NF-κB to promote proinflammatory cytokine production while suppressing IL-10 [66,67]. The inhibitor TFGF-18 alleviated neuroinflammation in LPS-treated microglia by suppressing NF-κB and related inflammatory mediators [66]. Dual inhibition of QC and GSK-3β blocks pE-Aβ production and tau hyperphosphorylation, providing a promising strategy for AD treatment [67].

In general, aberrant GSK-3β activity is linked to neurological, psychiatric, metabolic, neoplastic, and inflammatory diseases. As a key cellular signaling hub, GSK-3β converges major pathways including PI3K/Akt, Wnt/β-catenin and NF-κB. It responds to diverse stimuli and modulates cell survival, metabolism, proliferation, and inflammation. Dysregulated GSK-3β triggers distinct tissue-specific pathologies: tau hyperphosphorylation and synaptic damage in neurons, insulin resistance in pancreatic β-cells, elevated proinflammatory cytokines in immune cells, and enhanced proliferation and migration in tumor cells.

This central role of GSK-3β presents both therapeutic opportunities and potential liabilities. Systemic inhibition may induce off-target effects on multiple pathways, while selective targeting can concurrently intervene in various diseases and pathological processes. Strategies including dual-target inhibitors and tissue-specific delivery are designed to enhance efficacy and reduce systemic toxicity. Further clinical translation of GSK-3β modulators requires fuller insight into its universal roles in diseases and the development of highly selective molecular tools.

### 2.3. GSK-3β PET Imaging

Given GSK-3β’s key role in various diseases, its targeted PET tracers show broad application potential. PET imaging enables non-invasive, quantitative detection of both GSK-3β expression and enzymatic activity, supporting early diagnosis and therapeutic monitoring in AD, diabetes, inflammation, and cancer [30,35,60,66]. Specific binding to pathological regions in AD transgenic mice further underscores the diagnostic value of these tracers [68]. GSK-3β thus represents an ideal target for molecular neuroimaging. To date, GSK-3β inhibitors have advanced considerably, offering diverse scaffolds and SAR data for tracer design [69,70,71,72,73,74,75,76,77]. However, PET tracer development still faces major obstacles, including limited brain uptake, P-glycoprotein (P-gp) efflux, poor metabolic stability, and difficulties in confirming in vivo target specificity, and the intrinsic challenge of targeting an intracellular kinase, which demands that tracers cross both the cell membrane and the blood–brain barrier.

**Figure 2:**
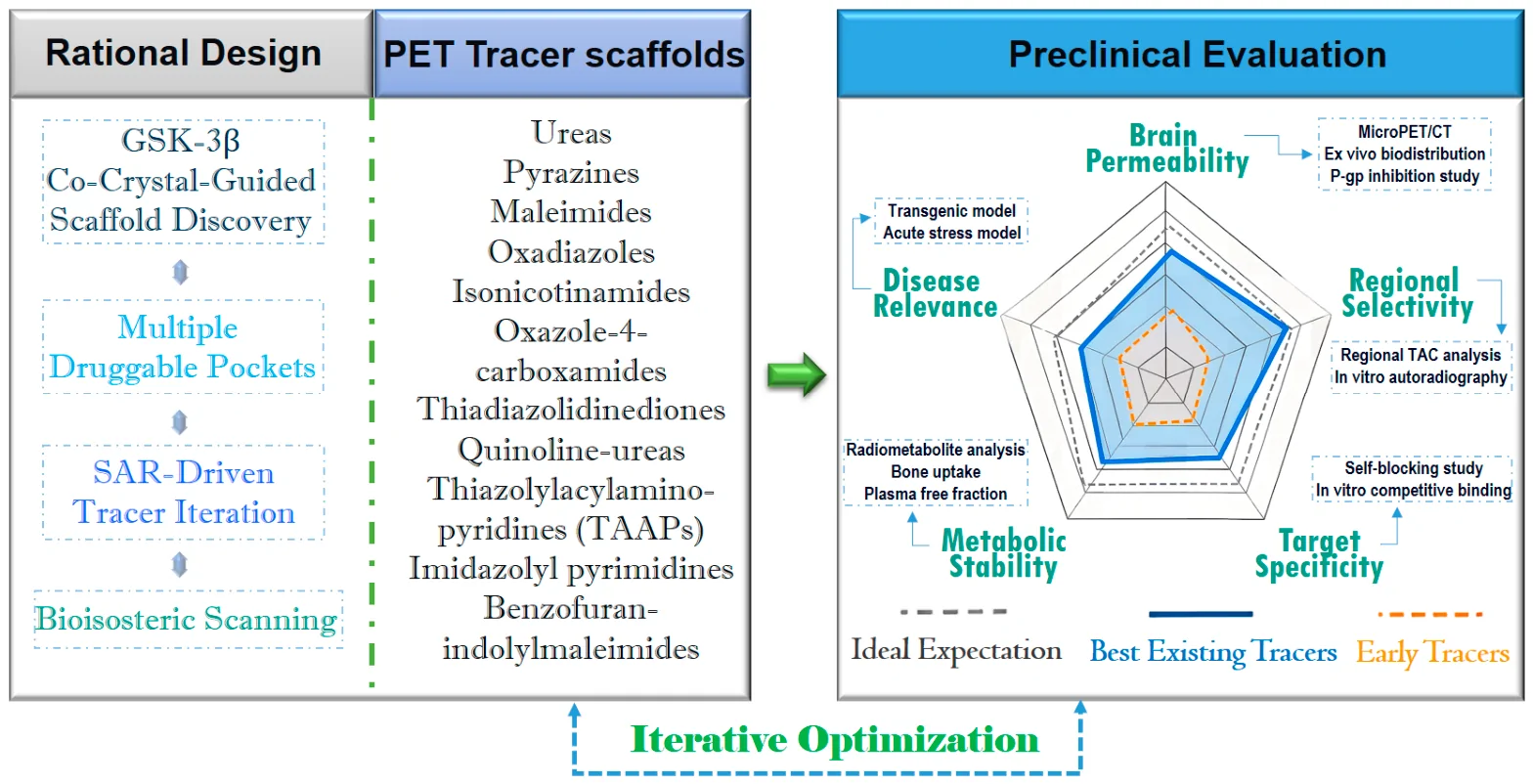
Rational design and preclinical evaluation of GSK-3β PET tracers. The radar schematic displays five preclinical indices: brain penetration, regional selectivity, target specificity, metabolic stability and disease relevance. Iterative optimization across these aspects helps obtain tracers with optimal in vivo profiles.

This review systematically surveys reported GSK-3β PET tracers by chemical scaffold, covering their design strategies, radiochemical syntheses, preclinical evaluation profiles, and key limitations. On this basis, this review discusses key challenges and proposes strategies, including multi-parameter optimization, tiered validation, and structure-guided design. These approaches facilitate rational development of high-performance tracers for AD mechanistic research and clinical use. Tracer scaffold classification, rational design, and preclinical evaluation are presented in Figure 2.

## 3. Classification of GSK-3β PET Tracers

Over the past two decades, GSK-3β PET tracers have been rapidly developed. Small-molecule inhibitors labeled with ^11^C or ^18^F have yielded tracers spanning 11 scaffold classes (Table 1). Research emphasis has shifted toward brain uptake, metabolic stability, and target specificity, with evaluation strategies expanding to transgenic models and nonhuman primates. Table 1 presents the key properties of representative tracers, providing a basis for structure–activity relationship analysis.

Table 1 summarizes the variations in affinity, brain uptake, and translational applicability of GSK-3β tracers across different scaffolds. The majority of these tracers exert nanomolar inhibitory activity. PF-367 (IC_50_ = 2.10 nM), A1070722 (*K_i_* = 0.60 nM), and the 10a-d analogs (IC_50_ = 1.30–5.80 nM) stand out with potent in vitro potency. Large discrepancies in brain uptake were noted: [^11^C]SB-216763 showed high brain uptake in rats and nonhuman primates (SUV = 1.90–2.50), in contrast to the limited brain penetration of [^11^C]AR-A014418 and [^11^C]PyrATP-1 [99]. [^18^F]10a–d showed heterogeneous regional distribution in rodents, and [^11^C]A1070722 in nonhuman primates mirrored the native GSK-3β distribution pattern. While early work relied on normal rodents, transgenic models and nonhuman primates are now widely adopted. [^18^F]F-CNPI has been validated in Thy1-GSK-3β transgenic mice, and [^11^C]OCM-44 as well as [^18^F]OCM-50 have undergone full pharmacokinetic characterization and blocking assays in nonhuman primates. Subsequent chapters summarize each tracer’s design, synthesis, radiolabeling, preclinical results, and limitations.

### 3.1. Ureas

**Figure 3:**
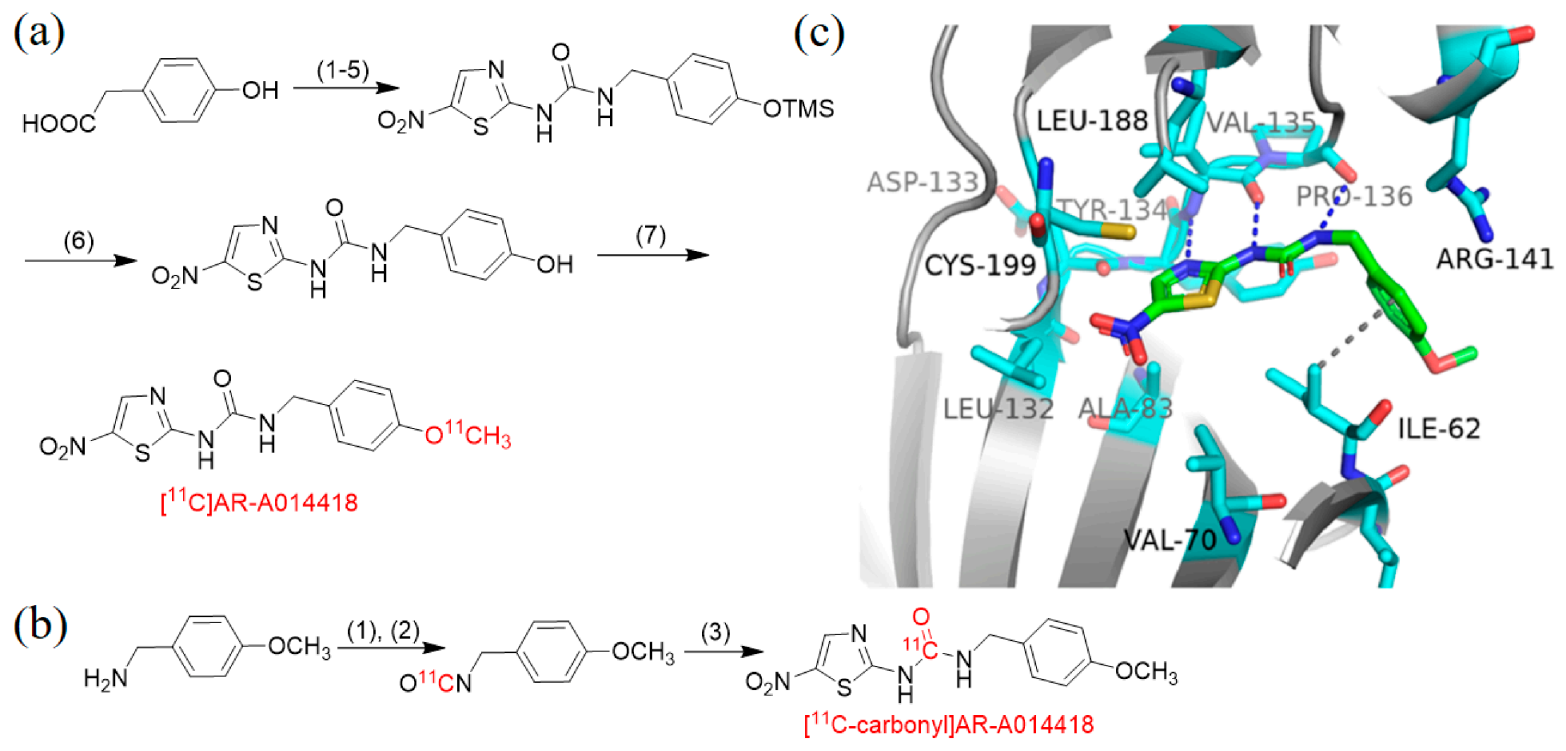
The synthesis of [^11^C]AR-A014418 (**a**) and [^11^C-carbonyl]AR-A014418 (**b**), together with AR-A014418 bound to the ATP-binding site of GSK-3β (PDB ID: 1Q5K [79]; interaction diagram generated using PyMOL [23] and PLIP [99]) (**c**). Route (**a**): (1) Trimethylsilyl chloride, Et_3_N, toluene, reflux; (2) Thionyl chloride, CH_2_Cl_2_; (3) Trimethylsilyl azide, 1,4-dioxane, r.t.; (4) reflux; (5) 5-nitrothiazol-2-amine, reflux; (6) HCl; (7) ^11^CH_3_I, Tetrabutylammonium hydroxide, DMF. Route (**b**): (1) BEMP, [^11^C]CO_2_, r.t; (2) POCl_3_, r.t; (3) 5-nitrothiazol-2-amine, r.t. [78,79].

[^11^C]AR-A014418, the first GSK-3β PET tracer, was synthesized via the [^11^C]methylation of a methoxy precursor [78] (Figure 3). [^11^C]AR-A014418 was synthesized by *O*-[^11^C]methylation of a deprotected hydroxyl precursor, which was prepared from an acyl chloride intermediate via Curtius rearrangement to an isocyanate, followed by coupling with 5-nitrothiazol-2-amine [78]. The carbonyl-labeled analog was prepared by [^11^C]CO_2_ fixation [79]. Despite a favorable LogP of 2.44, this tracer showed poor brain uptake (0.08%ID/g) in rats [76], and subsequent re-evaluation determined its *K_i_* against GSK-3β to be 770 nM, indicating weak binding affinity [79]. The co-crystal structure of AR-A014418 bound to GSK-3β (Figure 3c) shows the ligand occupying the ATP-binding pocket, with its thiazole-urea core forming hydrogen bonds with Val135 and the phenyl ring engaging in σ-π interactions with Ile62 [100]. Given its submicromolar affinity and poor brain penetration, this tracer is included here only as a historical starting point.

### 3.2. Pyrazines

**Figure 4:**
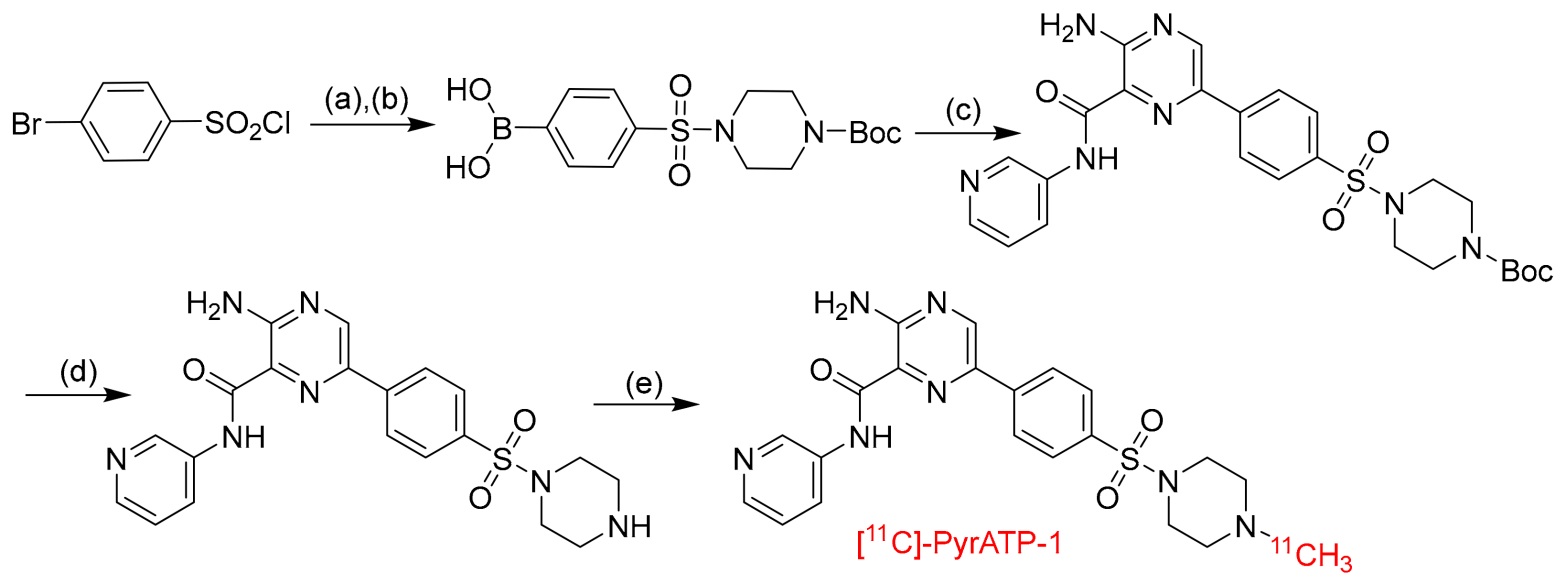
The synthesis of [^11^C]-PyrATP-1. (a) tert-butyl piperazine-1-carboxylate, Benzene; (b) Triisopropyl borate, THF, −78 °C; then n-Butyllithium, −78 °C; H_2_O; (c) 3-amino-6-bromo-*N*-(pyridin-3-yl)pyrazine-2-carboxamide, Pd(dppf)Cl_2_, Na_2_CO_3_, Toluene/Ethanol; (d) Trifluoroacetic acid, CH_2_Cl_2_; then Na_2_CO_3_; (e) [^11^C]CH_3_OTf, DMF [80].

Additionally, [^11^C]PyrATP-1 was developed as a ^11^C-labeled pyrazine-based GSK-3β inhibitor (*K_i_* = 4.9 nM) [80] (Figure 4). The piperazine precursor was prepared from a bromoarene via boronation, Suzuki coupling with a 2-aminopyrazine derivative, and Boc deprotection. *N*-[^11^C]methylation with [^11^C]CH_3_OTf under basic conditions afforded [^11^C]PyrATP-1 in a non-decay-corrected radiochemical yield (RCY) of 1–2%. Ex vivo autoradiography demonstrated specific binding in GSK-3β-rich rat brain regions, whereas in vivo PET imaging showed negligible brain uptake in rats and NHPs. Pretreatment with cyclosporine A elevated brain uptake (peak SUV ≈ 0.65), identifying [^11^C]PyrATP-1 as a P-gp substrate and highlighting the importance of efflux transporter screening in GSK-3β PET tracer development [80].

### 3.3. Maleimides

**Figure 5:**
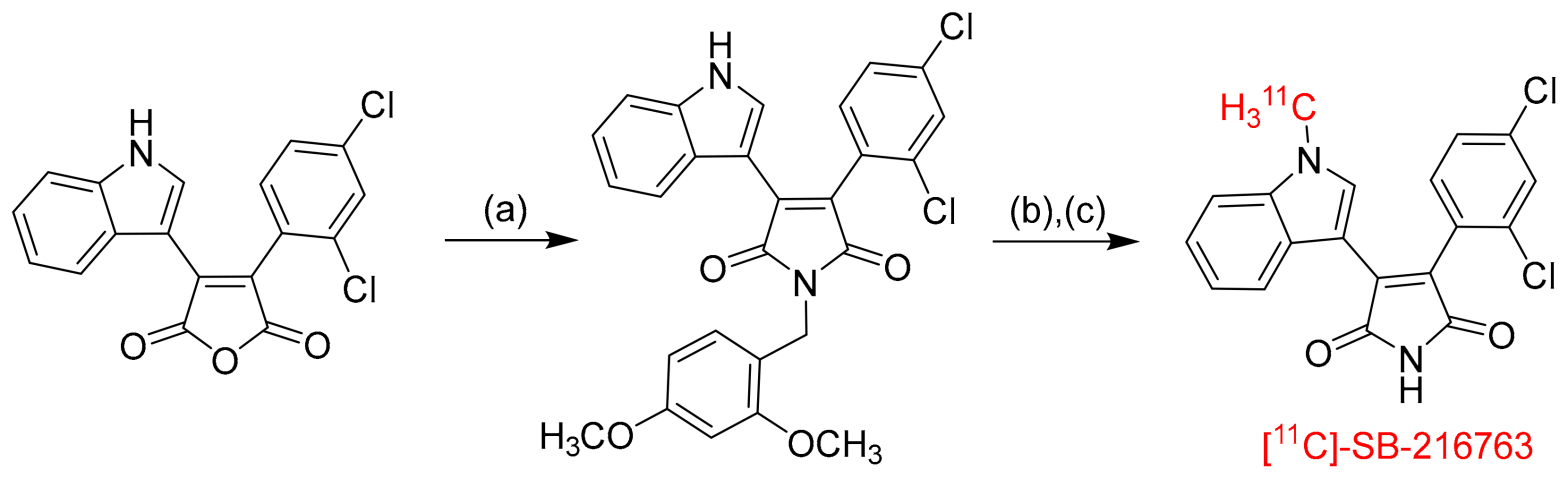
The synthesis of [^11^C]-SB-216763. (a) (2,4-dimethoxyphenyl)methanamine, AcOH, 140 °C; (b) NaH, [^11^C]CH_3_I, THF, 45 °C; (c) 50% CH_3_SO_3_H, THF, 110 °C [81].

In a further advancement, [^11^C]SB-216763 became the first GSK-3β PET tracer to cross the blood–brain barrier [81] (Figure 5). Radiosynthesis proceeded via cyclization of a dimethoxylated arylamine, followed by *N*-[^11^C]methylation with [^11^C]CH_3_I and deprotection, yielding [^11^C]SB-216763 with a non-decay-corrected RCY of 1% (based on [^11^C]CO_2_). Docking studies revealed key hydrogen bonds with the hinge region and π-cation interactions with Arg141 [101]. In vivo evaluation demonstrated high initial brain uptake (SUV = 2.50 at 3 min in rats; 1.90 at 5 min in NHPs), with higher uptake in GSK-3β-rich regions, while blocking data remain unreported [81].

**Figure 6:**
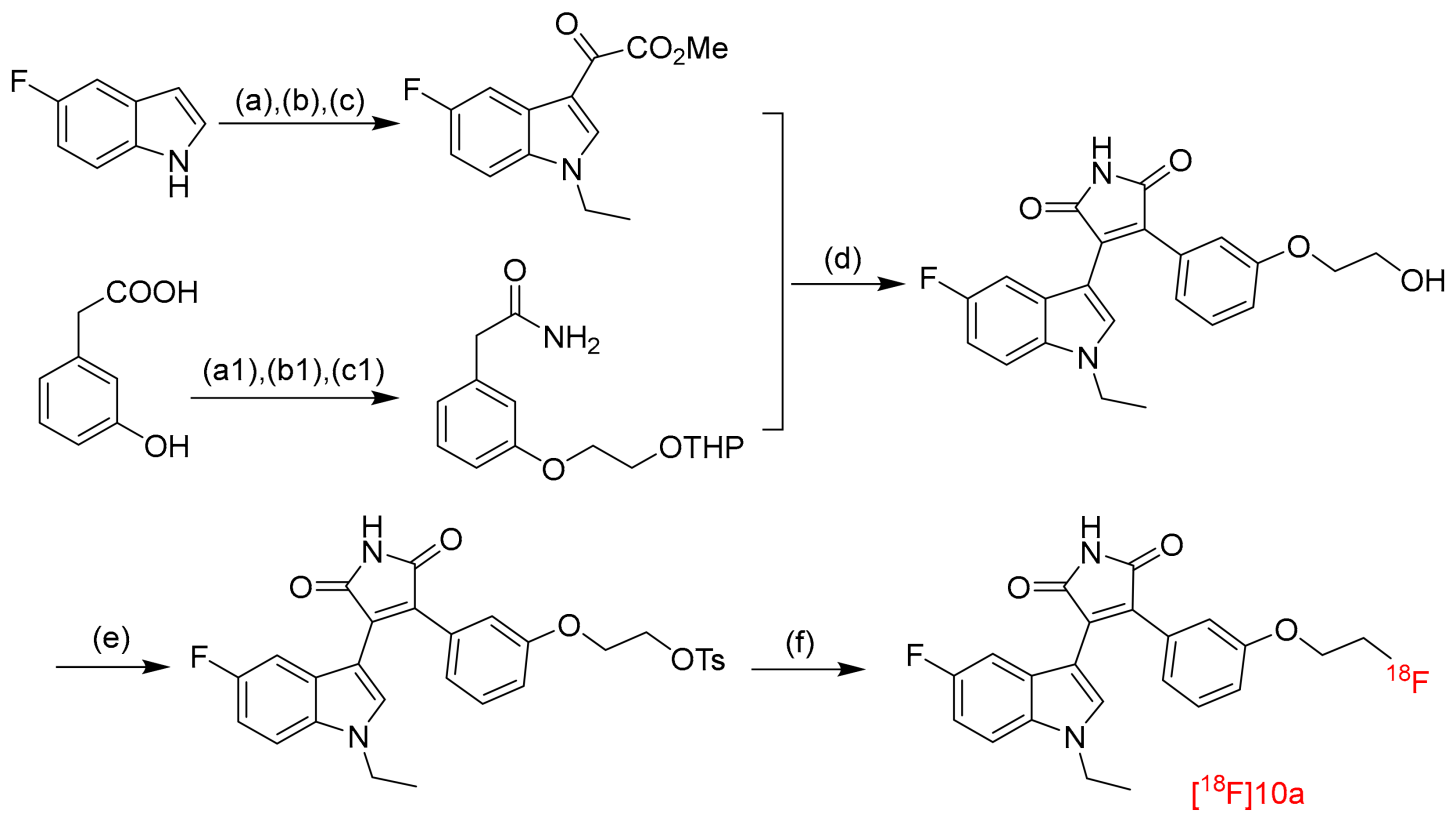
The synthesis of [^18^F]10a. (a) Oxalyl chloride, Et_2_O, 0 °C; (b) NaOMe, MeOH, −78 °C; (c) Bromoethane, NaH, DMF, 0 °C; (a1) MeOH, H_2_SO_4_, reflux; (b1) NH_3_(aq), rt; (c1) 2-(2-bromoethoxy)tetrahydro-2*H*-pyran, K_2_CO_3_, NaI, DMF, 60 °C; (d) Potassium tert-butoxide, THF, 0 °C; then HCl, r.t.; (e) *p*-Toluenesulfonyl chloride, Pyridine, 0 °C; (f) [^18^F]KF/K_222_, CH_3_CN, 90 °C [82].

From the same scaffold, [^18^F]10a was subsequently developed [82] (Figure 6). The precursor was prepared from an indole derivative through activation, *N*-ethylation, hydroxyethylation, cyclization, OTHP deprotection, and tosylation, followed by [^18^F]fluorination to afford [^18^F]10a in 36% radiochemical yield. PET imaging in rats showed peak brain uptake >0.4%ID/cc, while blocking studies showed no significant reduction [82].

**Figure 7:**
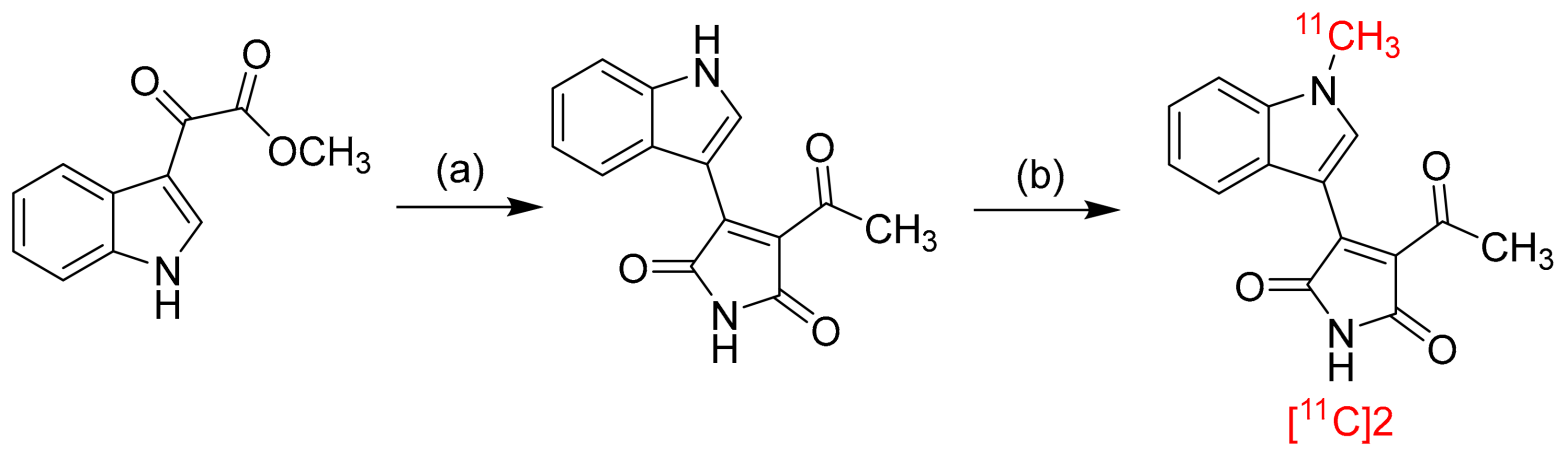
The synthesis of [^11^C]2. (a) 3-oxobutanamide, Potassium tert-butoxide, THF, −60 °C; (b) [^11^C]CH_3_I, Cs_2_CO_3_, DMF, 80 °C [83].

More recently, another maleimide-based GSK-3 tracer, [^11^C]2, was reported [83] (Figure 7). Its precursor was prepared via cyclization of acetoacetamide with methyl 3-indoleglyoxylate using potassium tert-butoxide, followed by [^11^C]methylation with [^11^C]CH_3_I to afford [^11^C]2 in 31 ± 4% decay-corrected RCY [83]. [^11^C]2 exhibited good brain uptake (2.93%ID/g) in normal mice and partially blockable binding, suggesting specific GSK-3 interaction. In 3×Tg-AD transgenic mice, amygdala uptake at 14 months was significantly elevated, consistent with increased GSK-3β expression in late-stage AD [83].

### 3.4. Oxadiazoles

**Figure 8:**
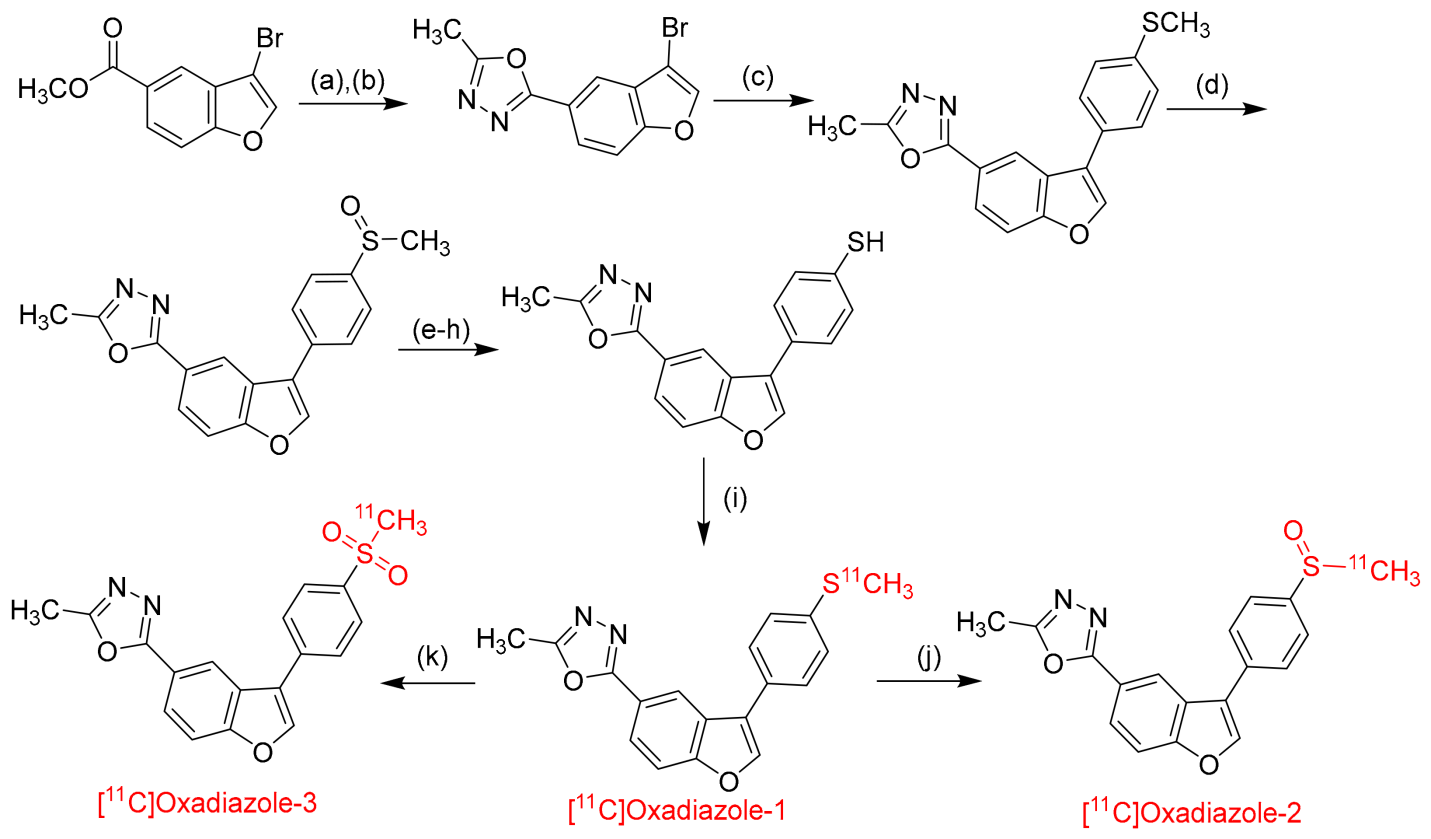
The synthesis of [^11^C]Oxadiazole-1, -2, and -3. (a) Hydrazine hydrate, MeOH, reflux; (b) Triethyl orthoacetate, 120 °C; (c) (4-(methylthio)phenyl)boronic acid, Pd(PPh_3_)_4_, Na_2_CO_3_, DME-H_2_O, reflux; (d) m-CPBA, CH_2_Cl_2_, 0 °C; (e) Trifluoroacetic anhydride, 2,6-lutidine, CH_2_Cl_2_, r.t.; (f) 1 M HCl, CH_3_OH, r.t.; (g) butyryl chloride, Et_3_N, CH_2_Cl_2_, r.t.; (h) 1 M NaOH, CH_3_OH, r.t.; (i) [^11^C]CH_3_I, NaOH, CH_3_OH, r.t. to give [^11^C]Oxadiazole-2; (j) CH_3_OH-H_2_O, 50 °C to give [^11^C]Oxadiazole-1; (k) CH_3_OH-H_2_O, 80 °C to give [^11^C]Oxadiazole-3 [84].

As an alternative strategy, [^11^C]Oxadiazole-1, -2, -3 were constructed based on the 1,3,4-oxadiazole scaffold. [84,102] Stepwise oxidation of the sulfur atom (thioether → sulfoxide → sulfone) was applied to modulate their physicochemical and biological properties [84] (Figure 8). The thiophenol precursor was prepared from a benzofuran methyl ester via hydrazinolysis, cyclization, Suzuki coupling, and subsequent functional group interconversions. [^11^C]Methylation gave [^11^C]Oxadiazole-1 (52 ± 5% RCY), which was further oxidized with Oxone^®^ to yield [^11^C]Oxadiazole-2 and [^11^C]Oxadiazole-3.

This unified approach allowed direct evaluation of how sulfur oxidation states affect brain uptake and clearance. The three tracers displayed nanomolar GSK-3β affinity (IC_50_ = 35–66 nM, Oxadiazole-1-3). In mice subjected to acute cold-water stress, [^11^C]Oxadiazole-1 and -3 showed roughly twofold higher brain uptake and area under the curve (AUC) with slower clearance versus controls, while [^11^C]Oxadiazole-2 showed no obvious changes, confirming that minor structural alterations can profoundly impact in vivo behavior [84].

### 3.5. Isonicotinamides

**Figure 9:**
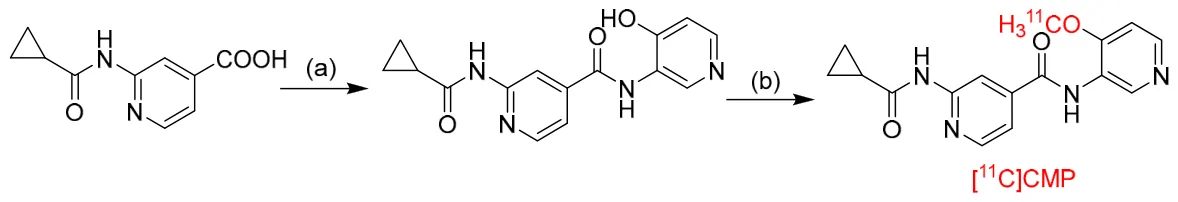
The synthesis of [^11^C]CMP. (a) 3-aminopyridin-4-ol, TPTU, DIPEA, CH_3_CN, r.t. (b) [^11^C]CH_3_I, NaOH, DMF [85].

Among the most investigated scaffolds, the isonicotinamide chemotype has evolved from early P-gp substrates to tracers validated in transgenic models. The early candidate [^11^C]CMP was synthesized via *O*-[^11^C]methylation of a phenolic precursor in 25 ± 5% decay-corrected RCY [85] (Figure 9). With IC_50_ = 3.4 nM (CMP) and LogP = 1.1, [^11^C]CMP showed high affinity but low brain uptake in rats. Cyclosporine A boosted its brain accumulation approximately 3-fold, confirming it as a P-gp substrate [85].

**Figure 10:**
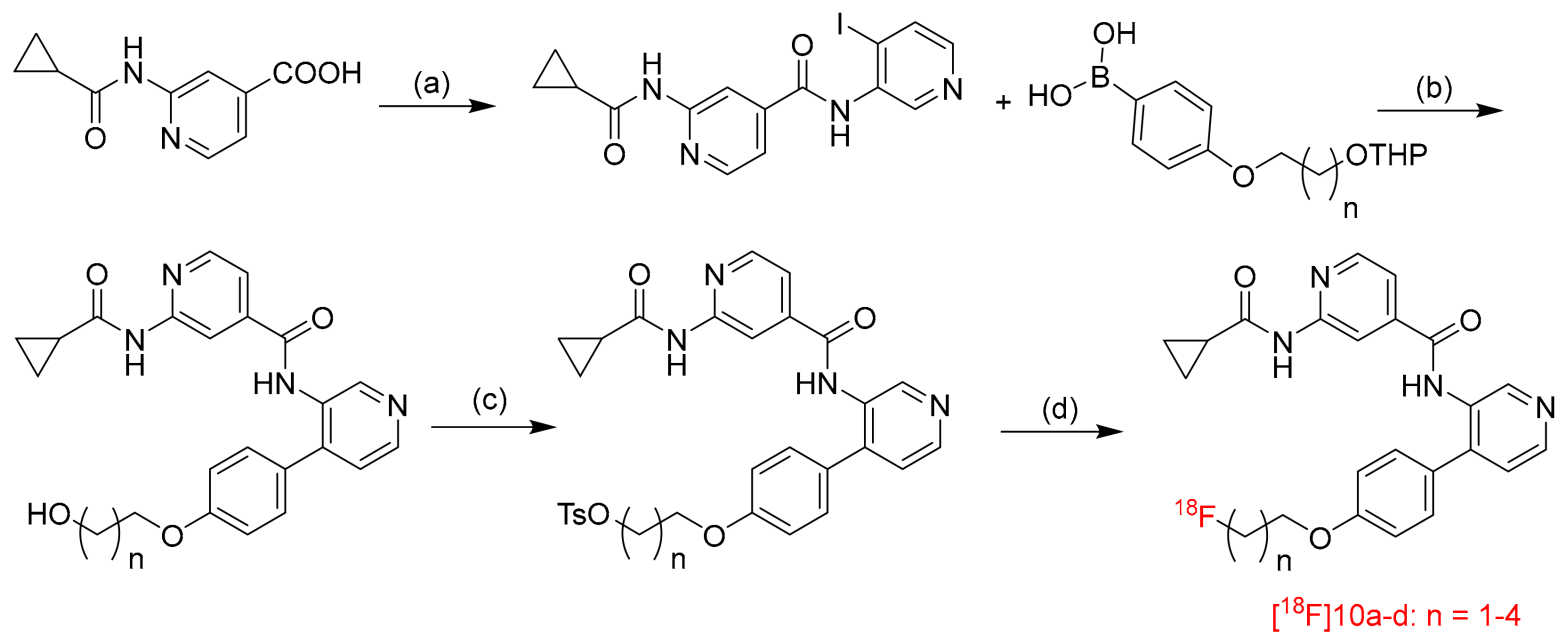
The synthesis of [^18^F]10a-d. (a) 4-iodopyridin-3-amine, EDCI, DMAP, DMF, r.t.; (b) Tetrakis(triphenylphosphine)palladium, K_2_CO_3_, dioxane, 130 °C; then HCl, r.t.; (c) *p*-Toluenesulfonyl chloride, DMAP, Et_3_N, CH_2_Cl_2_; (d) [^18^F]KF, K_222_, MeCN, 100 °C [86].

Further optimization led to the [^18^F]10a-d series, bearing fluoroalkoxy chains of increasing length (*n* = 1-4) [86] (Figure 10). The precursors were prepared via condensation, Suzuki coupling, and tosylation, followed by [^18^F]fluorination in 26–40% decay-corrected RCY. All four tracers showed potent GSK-3β affinity (IC_50_ = 1.30–5.80 nM, 10a-d). In vivo, the longer-chain derivatives [^18^F]10b-d displayed improved metabolic stability and heterogeneous regional distribution, with higher uptake in GSK-3β-rich regions such as the amygdala, cerebellum, and hippocampus [86].

**Figure 11:**
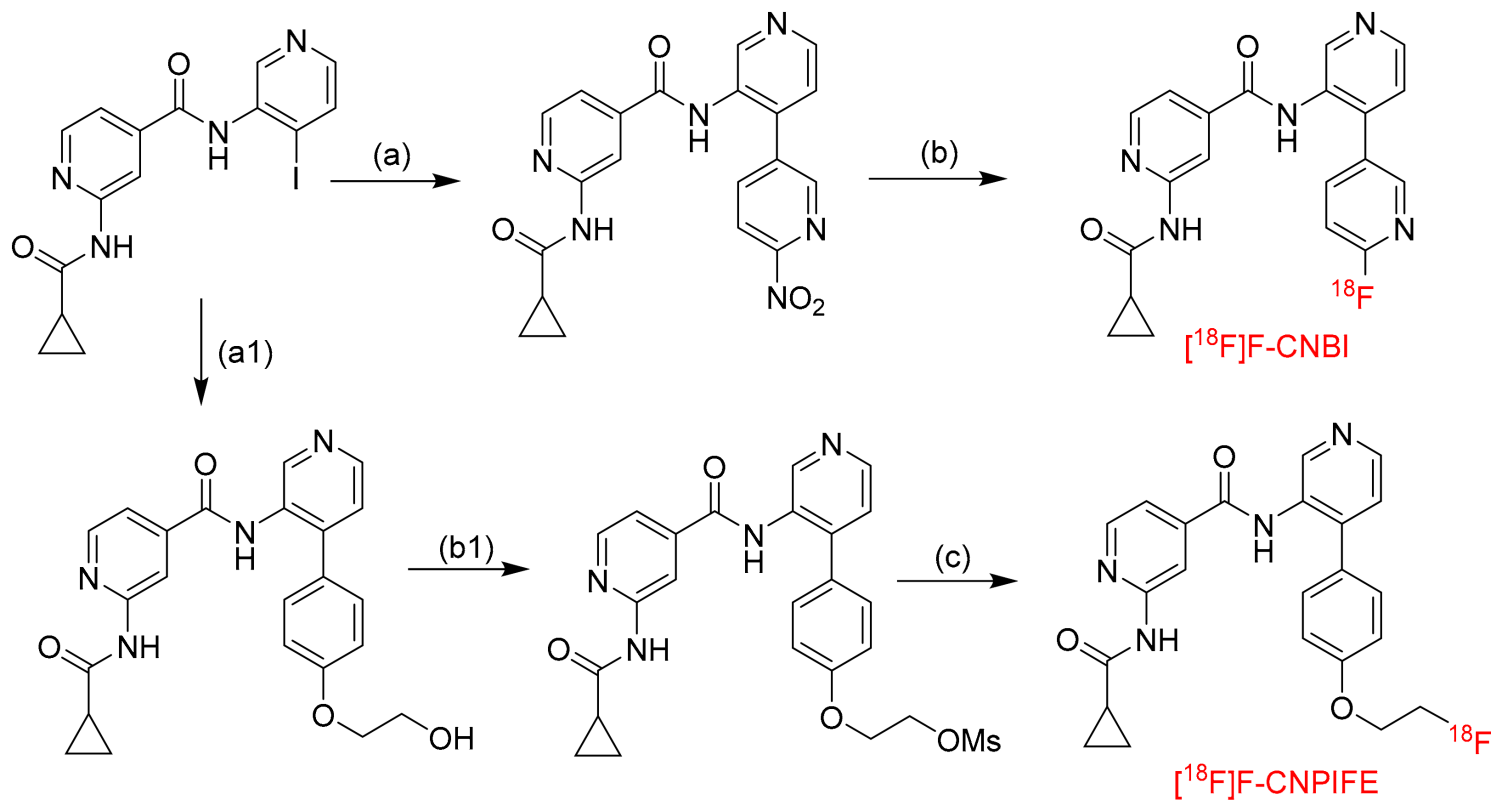
The synthesis of [^18^F]F-CNBI and [^18^F]F-CNPIFE. (a) 4-Nitro pyridinylboronic acid, Tetrakis(triphenylphosphine)palladium, K_2_CO_3_, THF-H_2_O, 80 °C; (b) K_222_/K_2_CO_3_, ^18^F^−^, 18-Crown-6, DMSO, 165 °C; (a1) (4-(2-hydroxyethoxy)phenyl)boronic acid, Tetrakis(triphenyl-phosphine)palladium, K_2_CO_3_, THF-H_2_O, 80 °C; (b1) methanesulfonic anhydride, THF; (c) K_222_/ K_2_CO_3_, ^18^F^−^, DMSO, 125 °C [87].

Subsequently, Stein et al. developed and compared two closely related isonicotinamide tracers, [^18^F]F-CNBI and [^18^F]F-CNPIFE [87] (Figure 11). [^18^F]F-CNBI was synthesized via Suzuki coupling with 4-nitropyridineboronic acid followed by direct aromatic [^18^F]fluorination, while [^18^F]F-CNPIFE was prepared using 4-(2-hydroxyethoxy)phenylboronic acid, mesylation, and radiofluorination. In vitro assays confirmed that [^19^F]F-CNPIFE was approximately 5-fold more potent than [^19^F]F-CNBI against both GSK-3α and GSK-3β (IC_50_ ≈ 19.40 nM vs 105.30 nM for both isoforms) and exhibited favorable PAMPA permeability similar to propranolol. Its ^18^F-labeled counterpart achieved 9.5-fold higher brain uptake in FVB/NJ mice at 30 min compared with [^18^F]F-CNBI. In contrast, blocking studies showed paradoxical results: co-injection of unlabeled ligand increased [^18^F]F-CNBI uptake by 2.5–3.0-fold, while [^18^F]F-CNPIFE showed only a mild increase (SUV 0.76 to 0.88) [87]. The authors attributed this to reduced peripheral organ retention [87,103].

**Figure 12:**
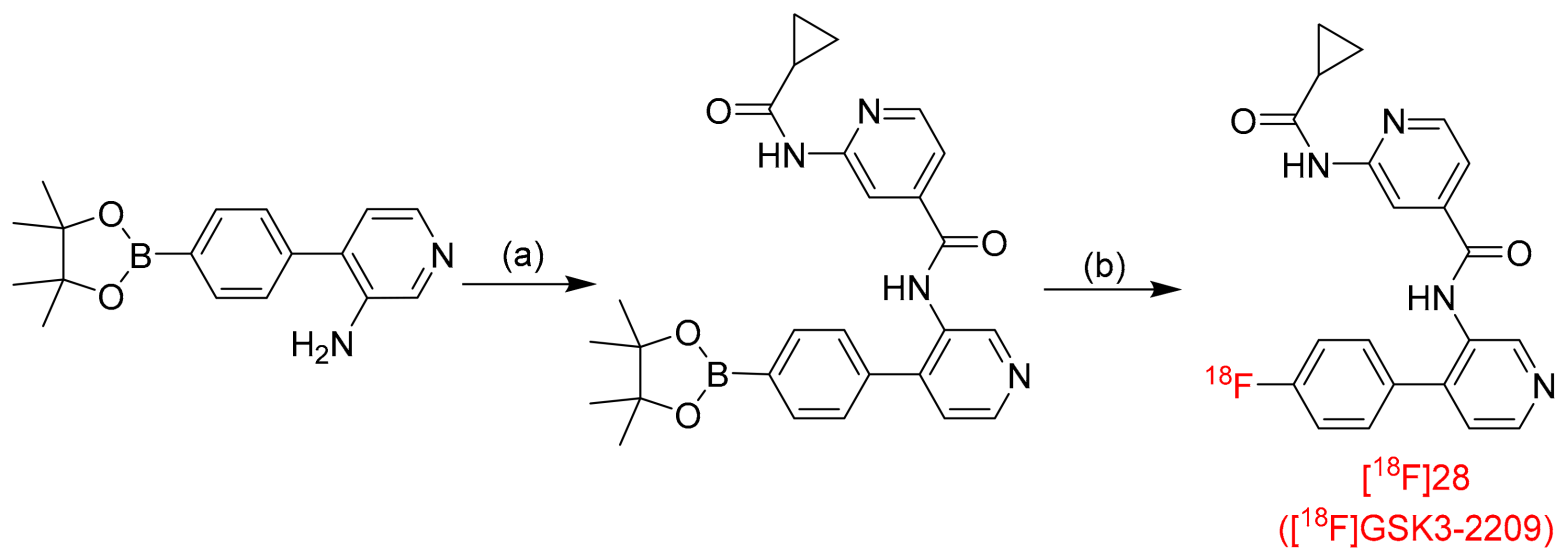
The synthesis of [^18^F]28 ([^18^F]GSK3-2209). (a) 2-(cyclopropanecarboxamido)isonicotinic acid, 2-Chloro-1-methylpyridinium iodide, Et_3_N, CH_2_Cl_2_; (b) [^18^F]TEAF, Cu(OTf)_2_(pyridine)_4_, DMAc/n-BuOH, 90 °C [88].

To address metabolic instability observed in earlier isonicotinamide tracers, [^18^F]28 ([^18^F]GSK3-2209) was designed with a C(sp^2^)–F motif [88] (Figure 12). The radiolabeling precursor was obtained by direct condensation of 2-(cyclopropanecarboxamido)isonicotinic acid with an arylboronic ester-substituted amine. Copper-mediated radiofluorination afforded [^18^F]28 in 32% decay-corrected RCY. In vitro, [^18^F]28 showed good rat plasma stability (>90% intact at 60 min) but faster metabolism in mouse than human liver microsomes (54% vs 89% intact). PET imaging displayed homogeneous brain distribution with approximately 32% blockable uptake [88].

**Figure 13:**
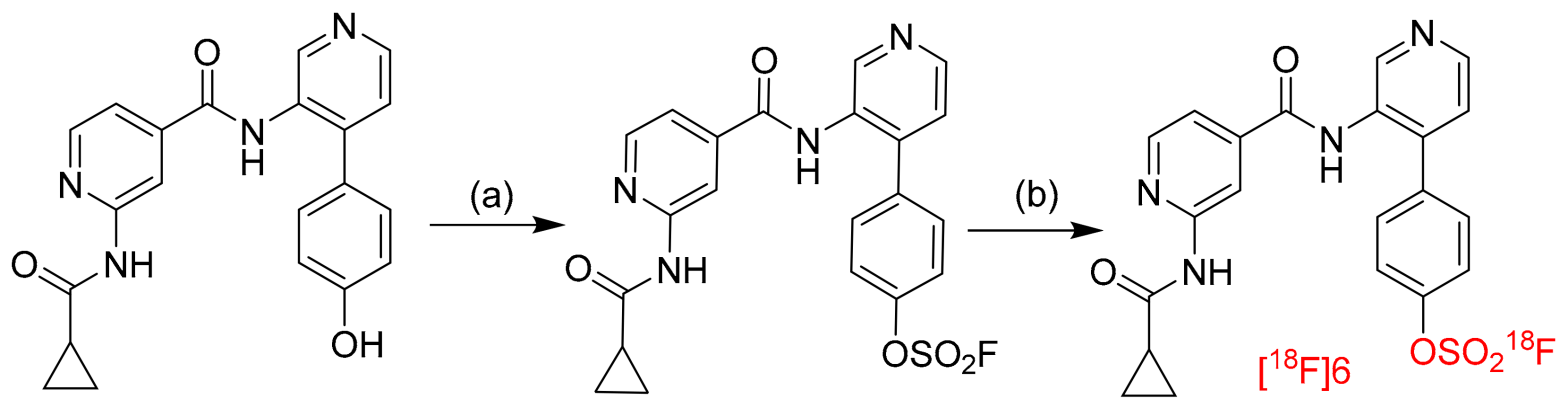
The synthesis of [^18^F]6. (a) 1-(fluorosulfonyl)-2,3-dimethyl-1*H*-imidazol-3-ium trifluoromethanesulfonate, Et_3_N, CH_3_CN, r.t.; (b) K_222_, ^18^F^-^, K_2_CO_3_, CH_3_CN, r.t. [89].

Further extending this chemotype, [^18^F]2 and [^18^F]6 were subsequently compared [89] (Figure 13). [^18^F]2, identical to [^18^F]28 and [^18^F]F-CNPI, was prepared via copper-mediated radiofluorination [88], while [^18^F]6 was synthesized via functional group interconversion and isotopic exchange in 26% decay-corrected RCY [88]. Both tracers displayed nanomolar GSK-3β affinity (2: IC_50_ = 24.29 nM; 6: IC_50_ = 43.99 nM). [^18^F]6 showed inferior BBB permeability and serum stability and was not further evaluated. By contrast, [^18^F]2 exhibited moderate brain uptake in mice (SUV = 0.92 at 5 min), but self-blocking paradoxically increased brain uptake (1.5–2-fold) while reducing liver retention, suggesting peripheral target saturation rather than specific cerebral binding [89].

Recently, [^18^F]F-CNPI (structurally identical to [^18^F]2 and [^18^F]28) became the first GSK-3β PET tracer validated in a GSK-3β-overexpressing transgenic mouse model [90]. This Thy1-driven GSK-3β (S9A) transgenic line exhibited 2.1–5.3-fold higher cerebral GSK-3β expression in late-stage (7.5-month-old) mice compared with wild-type controls. Brain uptake of [^18^F]F-CNPI exceeded that of [^18^F]F-CNBI across all groups and was highest in late-stage transgenic mice, with this difference further amplified by the P-gp inhibitor tariquidar. This dependence on efflux pump inhibition remains a key translational hurdle [90].

Overall, the development of isonicotinamide-based tracers follows a clear progression from early P-gp substrates ([^11^C]CMP) to structurally optimized mid-stage probes ([^18^F]10b-d, [^18^F]28) and recent candidates ([^18^F]2, [^18^F]F-CNPIFE, [^18^F]F-CNPI). Nevertheless, paradoxical blocking increases persist, and P-gp dependence remains a translational limitation for several members of this class [90].

### 3.6. Oxazole-4-Carboxamides

**Figure 14:**
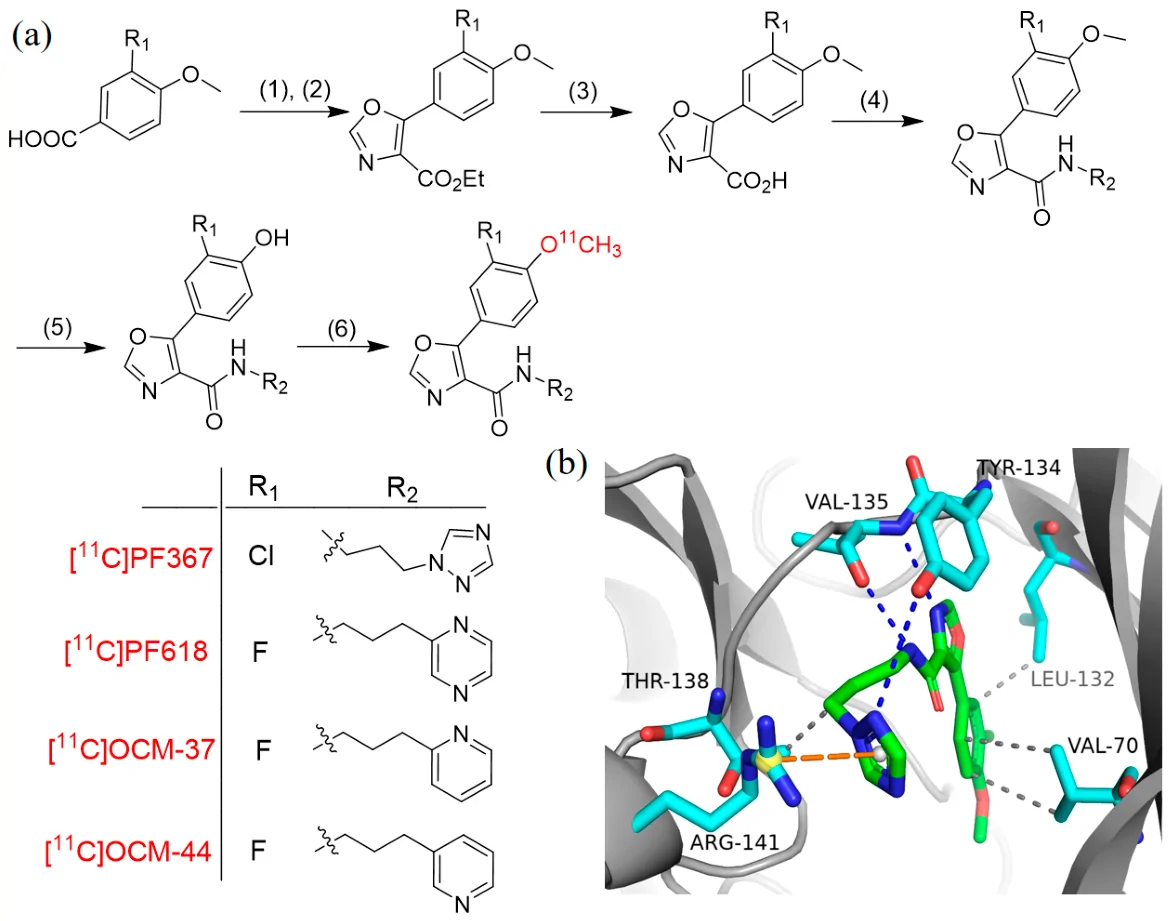
The synthesis of [^11^C]PF367, [^11^C]PF618, [^11^C]OCM-37, and [^11^C]OCM-44. (**a**) (1) Phosgene, cat. DMF, CH_2_Cl_2_, r.t.; (2) ethyl isocyanoacetate, Potassium tert-butoxide, THF, 0 °C; (3) NaOH, THF/H_2_O, r.t.; (4) R_2_-NH_2_, HATU, DIPEA, DMF, r.t.; (5) Boron tribromide, CH_2_Cl_2_, −78 °C; (6) [^11^C]CH_3_OTf, TBAOH, DMF, r.t. [22]. Co-crystals of PF-04802367 and GSK3β (PDB ID: 5K5N [22]) reveal PF-04802367 bound within the ATP pocket (**b**). The interaction diagram was generated using PyMOL [23] and PLIP [99].

The oxazole-4-carboxamide scaffold has been extensively investigated (Figure 14). [^11^C]PF-367 was synthesized by *O*-[^11^C]methylation of a phenolic precursor, which was prepared from a benzoic acid derivative via cyclization with ethyl isocyanoacetate, hydrolysis, amide condensation with 5-fluoropyridin-3-amine, and BBr_3_-mediated demethylation [22].

The co-crystal structure of PF-367 with GSK-3β (PDB: 5K5N) shows key binding interactions (Figure 14b) [22]. The oxazole and amide nitrogen form hydrogen bonds with Val135, while a C-H···O interaction with Asp133 enables isoform selectivity via the Asp-Glu switch [104], and a cation-π interaction occurs between the triazole and Arg141 [22]. Microscale thermophoresis (MST) analysis confirmed phosphorylation-independent binding [91,105,106,107]. In NHPs, [^11^C]PF-367 showed clear brain uptake (SUV = 1.00) with homogeneous distribution and specific binding confirmed by blocking studies [22].

As a close structural analog of [^11^C]PF-367, [^11^C]OCM-44 was synthesized via a similar route [91] (Figure 14). Knight et al. subsequently used [^3^H]PF-367 and [^11^C]OCM-44 to examine sex differences in GSK-3 expression [108]. In AD brains, GSK-3 density decreased in females but increased in males; in P301L mice, male transgenics showed elevated Bmax with no difference in females, and in vivo PET results were consistent with these findings. This study highlights sex as an important variable in GSK-3-targeted diagnostics and clinical trials [108].

Following this, Mossine et al. further evaluated [^11^C]OCM-44 in NHPs with full kinetic modeling and blocking studies [92]. PET imaging yielded Vτ values of 1.4–2.9 mL/cm^3^, consistent with human brain GSK-3 distribution, and PF-367 blocking achieved >80% target occupancy. However, Vτ increased over time, and plasma radiometabolites suggested brain-penetrant metabolites [92].

**Figure 15:**
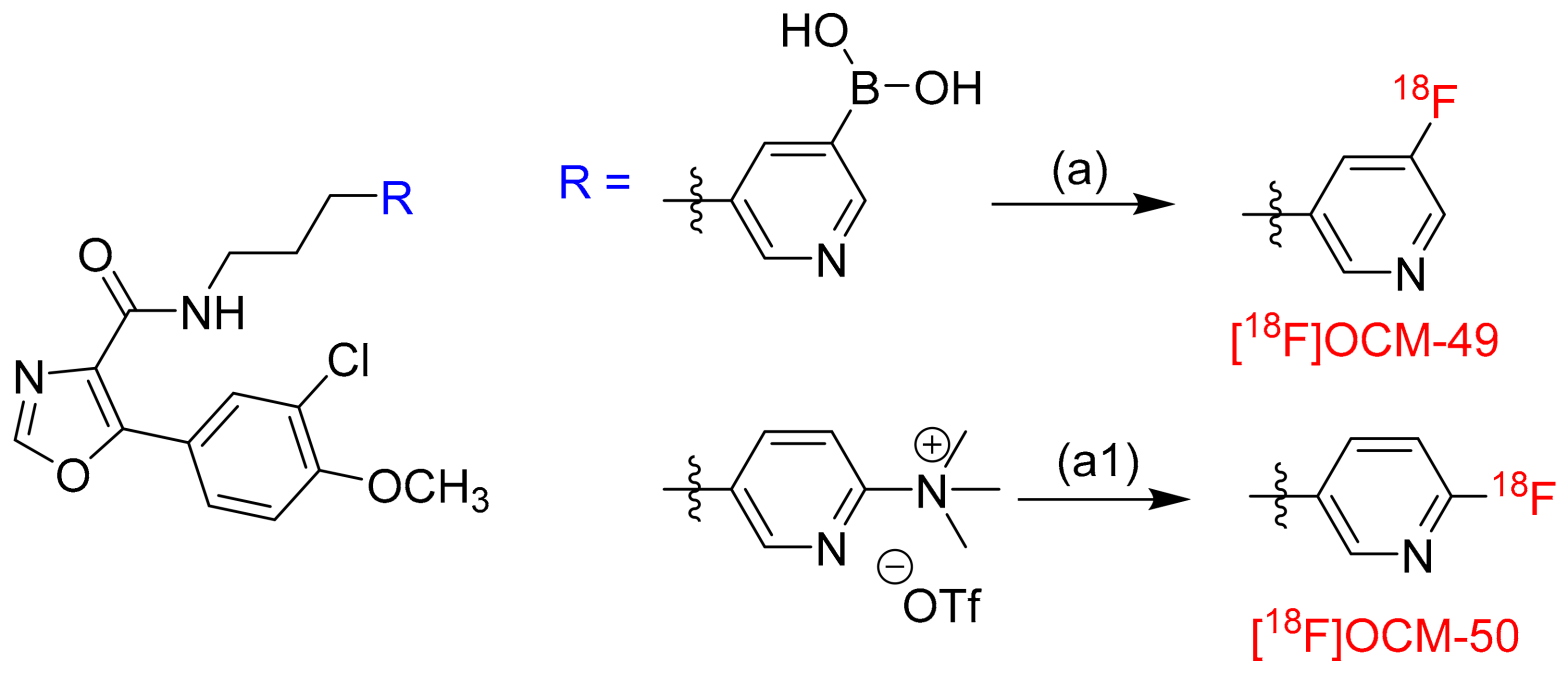
The synthesis of [^18^F]OCM-49 and [^18^F]OCM-50. (a) Cu(OTf)_2_, [^18^F]CsF, pyridine, DMF, 120 °C; (a1) [^18^F]KF/K_222_, CH_3_CN, 120 °C [93].

In parallel, two ^18^F-labeled analogs, [^18^F]OCM-49 and [^18^F]OCM-50, were developed using distinct radiolabeling strategies (Figure 15) [93]. [^18^F]OCM-49 was obtained in only 0.1% non-decay-corrected RCY via Cu(OTf)_2_-catalyzed radiofluorination. In contrast, [^18^F]OCM-50, obtained in 6.6% yield via [^18^F]KF, became the first ^18^F-labeled GSK-3β tracer to undergo full kinetic validation in NHPs [92]. OCM-50 inhibited GSK-3β by over 93% at 1 μM. In SD rats, peak brain uptake of [^18^F]OCM-50 reached an SUV of 2.0, double that of [^11^C]PF-367 [93]. In rhesus monkeys, it exhibited a peak brain SUV of 1–2, a plasma free fraction (f_p_) of 0.6%, and a stable Vτ of 0.39–0.87 mL/cm^3^, with target occupancy exceeding 80% in blocking studies, though the low f_p_ and potential off-target binding remain concerns [92].

In brief, the oxazole-4-carboxamide scaffold has yielded the most extensively validated GSK-3β PET tracers. [^11^C]PF-367 enables phosphorylation-independent total GSK-3β imaging, while [^11^C]OCM-44 and [^18^F]OCM-50 have undergone full kinetic validation in NHPs with high target occupancy and improved brain uptake, though brain-penetrant metabolites, low f_p_, and potential off-target binding remain limitations.

### 3.7. Thiadiazolidinediones

**Figure 16:**
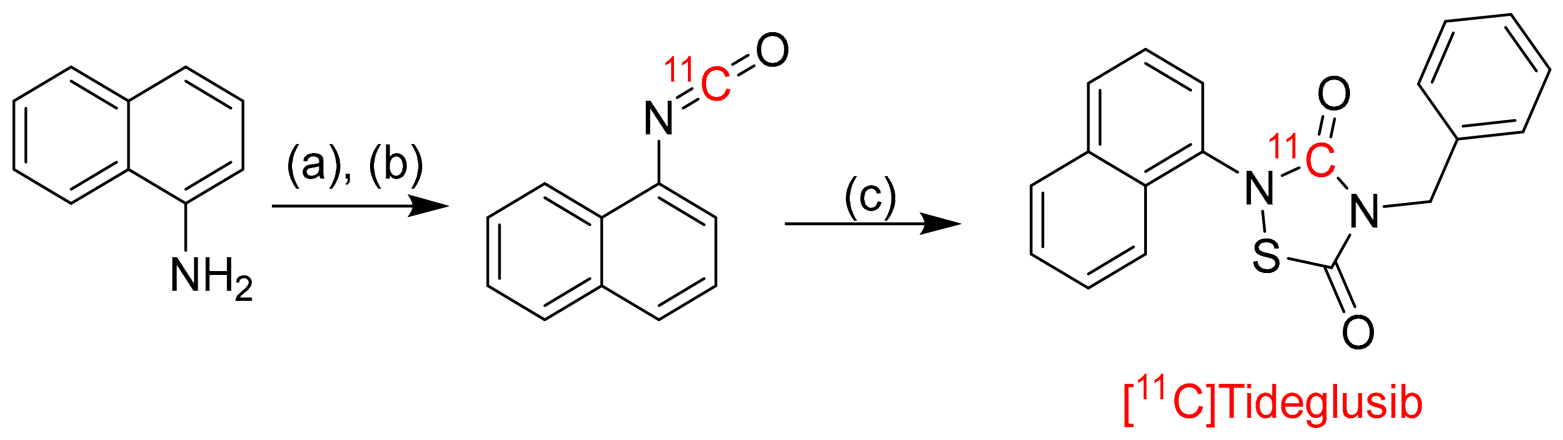
The synthesis of [^11^C]Tideglusib. (a) [^11^C]CO_2_, BEMP, CH_3_CN; (b) Phosphoryl chloride, THF/CH_3_CN; (c) (isothiocyanatomethyl)benzene, *N*-Chlorosuccinimide / Phosphoryl chloride; then H_2_O/Air [94].

Tideglusib, a thiadiazolidinedione-based irreversible GSK-3β inhibitor, advanced to clinical trials for AD [109,110,111]. [^11^C]Tideglusib was synthesized via [^11^C]CO_2_ fixation and cyclization in 2% non-decay-corrected RCY [94] (Figure 16). Rat PET imaging showed a peak brain uptake of SUV ≈ 0.70 followed by washout, a pattern inconsistent with the expected retention of an irreversible inhibitor, possibly attributable to low specific activity, rapid metabolism, or in vitro–in vivo differences.

### 3.8. Quinoline-Ureas

**Figure 17:**
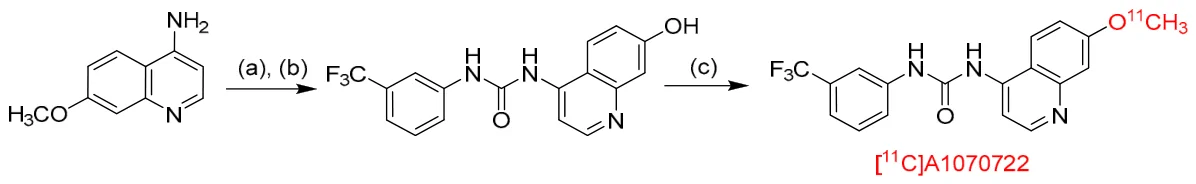
The synthesis of [^11^C]A1070722. (a) 6-(trifluoromethyl)pyridin-2-amine, triphosgene, CH_2_Cl_2_; (b) Boron tribromide, CH_2_Cl_2_; (c) NaOH, [^11^C]CH_3_I, DMF [95].

Subsequently, [^11^C]A1070722, a quinoline-urea-based GSK-3 tracer (*K_i_* = 0.6 nM, A1070722 ), was synthesized via *O*-[^11^C]methylation of its BBr_3_-demethylated precursor in 40 ± 5% radiochemical yield [95] (Figure 17). In vervet monkeys, it displayed heterogeneous uptake consistent with endogenous GSK-3 distribution, highest in the frontal cortex and lowest in the caudate, putamen, and thalamus. Uptake failed to reach equilibrium within 90–120 min, and the Vτ was low, underscoring the need for ^18^F-labeled tracers.

### 3.9. Thiazolylacylaminopyridines

**Figure 18:**
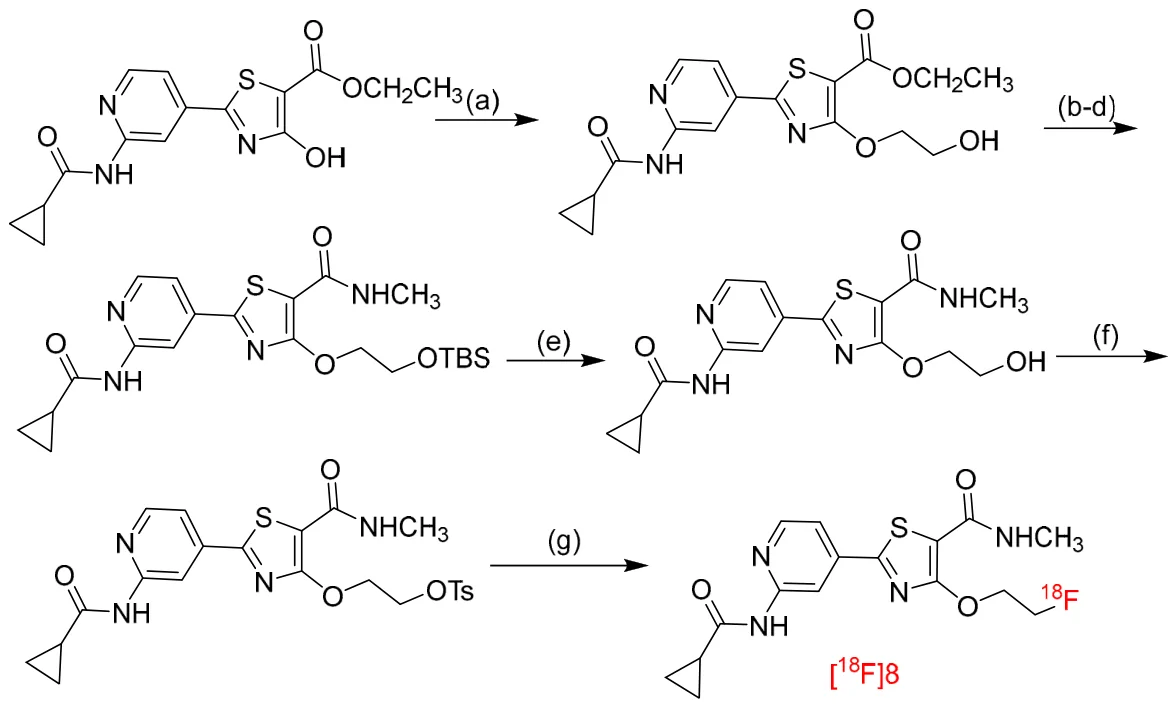
The synthesis of [^18^F]8. (a) 2-bromoethanol, Potassium tert-butoxide, DMF, 120 °C; (b) LiOH, MeOH/THF, 40 °C; (c) imidazole, TBSCl, DMF/THF, 40 °C; (d) CH_3_NH_2_·HCl, HATU, DIPEA, DMF, r.t.; (e) Tetrabutylammonium fluoride, THF, 40 °C; (f) *p*-Toluenesulfonyl chloride, Et_3_N, CH_2_Cl_2_, r.t.; (g) K_222_/K_2_CO_3_, ^18^F^−^, MeCN, 100 °C [96].

In another approach, [^18^F]8, a TAAP-based tracer, relies on an intramolecular hydrogen bond to stabilize its bioactive conformation [96] (Figure 18). The tosylate precursor was prepared via sequential hydroxyethylation and TBAF-mediated deprotection. Optimized [^18^F]fluorination with tetrabutylammonium bicarbonate improved the decay-corrected RCY from <5% to 22.3%. The tracer showed moderate lipophilicity (LogD_7.4_ = 2.24 ± 0.20), high metabolic stability (more than 95% intact in plasma at 4 h), and negligible in vivo defluorination. Brain uptake in KM mice was modest (1.68%ID/g at 2 min), presumably restricted by its high topological polar surface area (tPSA) and large number of rotatable bonds [96].

### 3.10. Imidazolyl Pyrimidines

**Figure 19:**
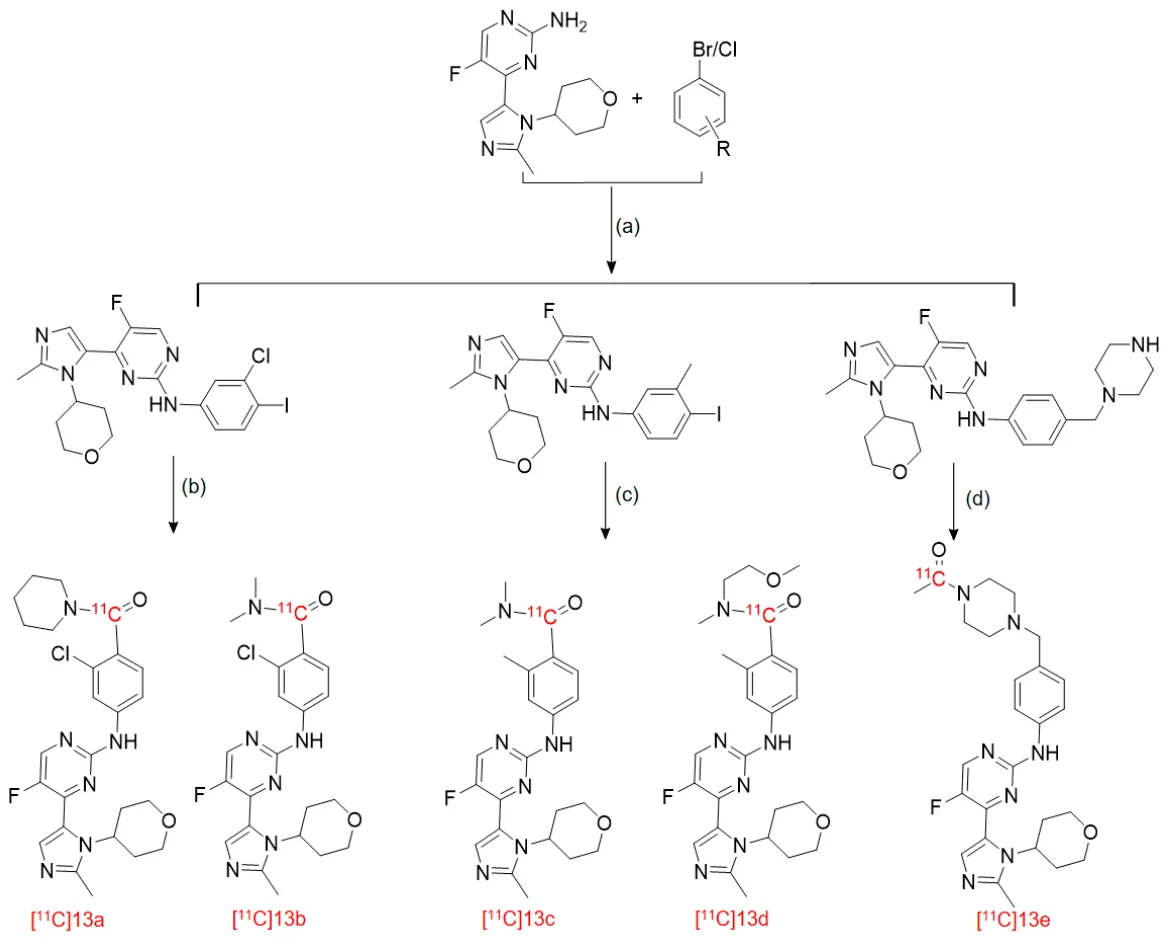
The synthesis of [^11^C]13a-e. (a) Tris(dibenzylideneacetone)dipalladium(0), xantphos, Cs_2_CO_3_, 1,4-dioxane, 90–100 °C; (b) [^11^C]CO, xantphos, [(Cinnamyl)PdCl]_2_/ Tetrakis(triphenyl-phosphine)palladium, piperidine/diethylamine; (for [^11^C]13a and [^11^C]13b); (c) [^11^C]CO, xantphos, [(Cinnamyl)PdCl]_2_, Diethylamine/(2-methoxy)methylamine; (for [^11^C]13c and [^11^C]13d); (d) [^11^C]CO, Tetrakis(triphenylphosphine)palladium, CH_3_I, THF, 100°C (for [^11^C]13e) [97].

In 2025, Li et al. reported five ^11^C-labeled imidazolyl pyrimidine-based tracers, [^11^C]13a-e [97], which were derived from the parent scaffold [112]. (Figure 19). The precursors were constructed via Buchwald–Hartwig coupling, and radiosynthesis was achieved through palladium-catalyzed [^11^C]CO carbonylation via three distinct routes, yielding decay-corrected RCY of 1.6–13.8% and radiochemical purity >99%. All five compounds showed high GSK-3β affinity (*K_i_* = 2.49–4.95 nM) and favorable CNS penetration parameters. Autoradiography with [^3^H]13e on rat brain sections revealed a heterogeneous and blockable binding pattern, while NHP PET imaging showed low brain uptake and rapid clearance for all five tracers. [^11^C]13e was identified as a P-gp substrate, with brain uptake markedly increased by Tariquidar pretreatment [97].

### 3.11. Benzofuran-Indolylmaleimides

**Figure 20:**
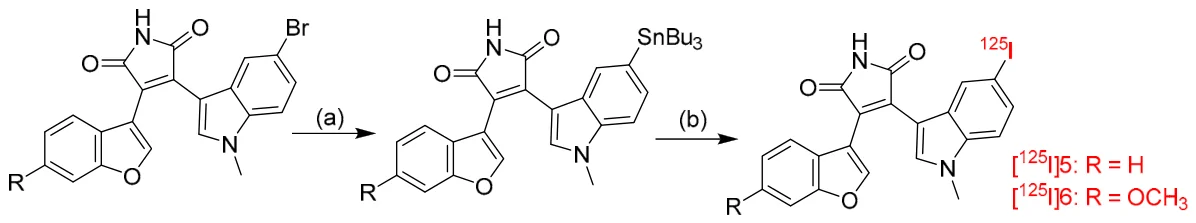
The synthesis of [^125^I]5 and [^125^I]6. (a) Hexabutylditin, Tetrakis(triphenyl-phosphine)palladium, Et_3_N, 1,4-Dioxane, reflux; (b) [^125^I]NaI, HCl, H_2_O_2_, r.t. [98].

[^125^I]5 and [^125^I]6 are the first potential SPECT imaging tracers based on the benzofuran-indolylmaleimide scaffold [98] (Figure 20). Synthesized via Stille coupling and iododestannylation, they showed initial brain uptake of 1.79 and 1.76%ID/g in ddY mice with favorable clearance and deiodination stability. This work expanded imaging modalities for GSK-3β and provided new lead structures for SPECT development.

The above survey of 11 scaffolds reveals recurrent obstacles that transcend individual chemotypes. In the following section, we systematically analyze these cross-scaffold challenges and propose a unified framework for future optimization.

## 4. Comparative Analysis of Tracers Across Scaffolds

### 4.1. Evolution of Design Strategies

Over the past two decades, the development of GSK-3β PET tracers has undergone a clear paradigm shift. The early phase (2005–2014) focused on simple ^11^C-methylation of known inhibitor scaffolds, generally using high affinity (*K_i_* < 10 nM) and moderate lipophilicity (LogP 2-3) as core screening criteria. However, the failures of AR-A014418 (*K_i_* = 770 nM, brain uptake 0.08%ID/g) [78] and PyrATP-1 [80] (*K_i_* = 4.9 nM but a P-gp substrate) demonstrated that affinity and LogP alone cannot ensure effective brain penetration.

The second phase (2015–2020) introduced P-gp efflux screening and metabolic stability assessment. [^11^C]SB-216763 [81] achieved high brain uptake (SUV ~2.50 in rats), but non-specific binding and lack of blocking data limited its utility. [^11^C]PF-367 [22] and the subsequent OCM series optimized isoform selectivity and brain exposure through structure-guided design, and performed full kinetic modeling in nonhuman primates (NHPs). This phase established P-gp avoidance and metabolite analysis as critical evaluation metrics.

The current third phase (2021-present) has revealed more insidious failure modes. Isonicotinamide-based tracers ([^18^F]2 [88,89], [^18^F]F-CNPIFE [87]) showed paradoxical increases in brain uptake upon blocking, indicating that peripheral target saturation can alter the plasma free fraction and produce false-positive signals. [^11^C]OCM-44 [91,92] was found to generate brain-penetrant radiometabolites, severely compromising quantification. These findings indicate that brain uptake and blocking studies alone are insufficient to validate target engagement; arterial input functions, metabolite correction, and low-affinity controls are essential.

### 4.2. Systematic Attribution of Key Limitations

#### 4.2.1. P-gp Efflux

Among the 11 scaffold classes, at least five (pyrazines, early isonicotinamides, imidazolyl pyrimidines, some isonicotinamides, and quinoline-ureas) are clearly or highly suspected P-gp substrates. Typical evidence includes a 2- to 5-fold increase in brain uptake after cyclosporine A or tariquidar pretreatment. Importantly, P-gp recognition does not correlate simply with affinity or LogP. For example, [^11^C]CMP [85] (IC_50_ = 3.40 nM (CMP), LogP = 1.10) exhibits pronounced efflux, illustrating that even high-affinity tracers with moderate lipophilicity can be P-gp substrates. Structural analysis suggests that multiple hydrogen-bond donors and a large topological polar surface area (tPSA) are common risk factors for P-gp recognition [96]; the modest brain uptake of [^18^F]8 further illustrates this point, as its high tPSA and numerous rotatable bonds both contribute to its limited brain penetration, indicating that multiple physicochemical parameters beyond affinity and lipophilicity influence brain uptake.

#### 4.2.2. Brain-Penetrant Metabolites

In NHPs, the Vτ of [^11^C]OCM-44 increased over time, and plasma metabolite analysis revealed the presence of less polar radioactive species capable of crossing the blood–brain barrier. This leads to systematic overestimation of target density, an issue that cannot be corrected by reference tissue models [91,92]. By contrast, [^18^F]OCM-50 generates mainly more polar fragments and no detectable brain-penetrant metabolites. However, its extremely low *f_p_* (0.6%) limits the amount of free tracer available for brain entry, resulting in low Vτ (0.39–0.87 mL/cm^3^) and suboptimal signal-to-noise ratios in low-expression regions [92,93]. These contrasting observations highlight a broader principle: rapid peripheral metabolism is not always detrimental. Indeed, rapid clearance of the parent compound can facilitate reversible binding kinetics and enable reliable quantification using equilibrium models, provided that (i) radiometabolites remain polar and do not cross the BBB, and (ii) the metabolic clearance rate is consistent across subjects. The key issue is not metabolic stability per se, but rather the absence of brain-penetrant radiometabolites [113,114].

#### 4.2.3. Paradoxical Blocking Increases

The isonicotinamide tracers [^18^F]2 [88,89], [^18^F]F-CNPIFE [87] and [^18^F]F-CNBI [87] all show a 1.5- to 3.0-fold increase in brain uptake after injection of the non-radioactive blocking agent. This counter-intuitive phenomenon can be explained as follows: a high-affinity blocker rapidly occupies GSK-3β in peripheral tissues (liver, muscle, etc.), reducing peripheral clearance of the tracer and thereby increasing the AUC of free tracer in plasma, which passively increases brain uptake. This artifact can be identified by co-injection of a low-affinity enantiomer (which should not alter plasma pharmacokinetics) or by direct measurement of plasma free fraction and parent compound AUC. No GSK-3β tracer has systematically excluded this possibility to date. Beyond these paradoxical increases, several tracers also lacked any reported blocking data. For example, the first brain-penetrant maleimide [^11^C]SB-216763 showed high initial brain uptake but had no reported blocking or specificity data [81], while [^18^F]10a from the same scaffold exhibited no significant reduction in blocking studies, suggesting predominantly non-specific binding [82]. These examples further underscore the importance of systematic blocking studies, including appropriate negative controls, for validating target specificity.

### 4.3. Multi-Dimensional Comparison of Representative Tracers

Based on the above analysis, representative tracers are compared from the perspective of readiness for clinical translation (Table 2):

Overall, [^18^F]OCM-50 [92,93] is the closest candidate to clinical application, but its low fp may limit signal in low-expression brain regions (e.g., white matter). [^11^C]OCM-44 [91,92] is well-validated in NHP quantification with demonstrated target occupancy, yet its brain-penetrant radiometabolites pose a major challenge for reliable quantification. [^11^C]PF-367 [22] is well validated but limited by the 20 min half-life of ^11^C. [^18^F]F-CNPI [90] first needs to resolve the origin of its paradoxical blocking behavior.

An additional consideration that has been largely overlooked in GSK-3β tracer development is the relationship between target density (B_max_) and tracer affinity, which in vivo determines the binding potential (BP_ND_ = f_ND_ × B_max_ / K_D_) [115,116]. Direct saturation binding studies have provided quantitative B_max_ estimates: 960 fmol/mg in mouse brain (*K_d_* = 13.02 nM) [91], 64.50–83.40 fmol/mg in rat hippocampus [97], and 349–1104 fmol/mg in human cortex [108]. These values consistently indicate a modest target density, of which GSK-3β is estimated to account for the majority (two- to four-fold higher density than GSK-3α) [91]. Combined with the best reported low-nanomolar tracer affinities (e.g., *K_d_* = 1.09–6.46 nM in rat hippocampus [97]) and low tissue free fractions (e.g., f_p_ = 0.6% for [^18^F]OCM-50 [92]), these B_max_ and *K_d_* values suggest a modest in vivo binding potential, which is well below the BP_ND_ values (≥ 5–10) generally considered necessary for reliable quantitative PET imaging [117]. These observations indicate that limited target density is a demonstrated constraint in regions with direct B_max_ measurements, but remains a hypothesis for regions and isoforms where such data are still lacking.

Across the representative tracers surveyed here, a consistent trend emerges: tracers that fail to address P-gp efflux or brain-penetrant metabolite formation have consistently stalled at the preclinical stage, whereas those with favorable profiles in these respects have advanced to NHP quantification. This empirical pattern underscores the importance of the early characterization of efflux and metabolic profile in future tracer development efforts.

It should be noted that many preclinical studies discussed in this review involved relatively small sample sizes. For example, NHP PET studies of [^11^C]OCM-44 and [^18^F]OCM-50 included only two animals per radiotracer [92], with initial [^18^F]OCM-50 NHP imaging performed in a single monkey [93]. Human post-mortem tissue studies using [^3^H]PF-367 and [^11^C]OCM-44 analyzed as few as 2–3 samples per group [108]. While these studies provide valuable proof-of-concept data, the limited sample sizes reduce statistical power and may affect generalizability. Larger cohorts are needed to validate these preliminary findings and support clinical translation.

### 4.4. Interspecies Differences in BBB Transport, Metabolism, and Target Expression

The clinical translation of GSK-3β PET tracers is significantly complicated by interspecies differences in BBB transport, metabolic profiles, and target expression. A critical evaluation of these discrepancies is essential to bridge the gap between preclinical data and human outcomes.

Firstly, regarding BBB transport and efflux mechanisms, while P-gp plays a dominant role in limiting brain penetration across species, its substrate specificity and expression levels can vary. The oxazole-4-carboxamide scaffold, exemplified by [^18^F]OCM-50, demonstrated higher brain uptake in rhesus monkeys than in rats, highlighting the necessity of NHP studies as a predictive model for human BBB penetration [92,93]. Conversely, tracers like [^11^C]PyrATP-1 exhibited species-independent P-gp substrate characteristics, as P-gp efflux inhibition increased brain uptake in both rats and NHPs [99]. These findings underscore that rodent data alone are insufficient to predict human brain exposure; validation in NHPs is crucial for assessing the translational potential of BBB penetration.

Secondly, significant metabolic differences exist, particularly in the liver. Many tracers show favorable metabolic stability in rodent plasma but undergo rapid degradation in human liver microsomes (HLM), or vice versa. For example, [^18^F]28 ([^18^F]GSK3-2209) exhibited high stability in rat plasma (> 90% intact) but demonstrated faster metabolism in mouse liver microsomes compared to HLM (54% vs. 89% intact, respectively) [88]. Such discrepancies can lead to the generation of brain-penetrant radiometabolites that confound quantitative accuracy in humans, even if not observed in rodent studies. Therefore, parallel metabolic screening in both rodent and human systems is mandatory during the optimization phase.

Lastly, GSK-3β expression and regulation differ across species and brain regions [95]. While the overall distribution pattern of GSK-3β is conserved, its density and activity, such as phosphorylation states, can vary. For instance, sex differences in GSK-3β expression observed in Alzheimer’s disease (AD) models, where male transgenics showed elevated B_max_ while females did not, highlight the complexity of translating findings from animal models that may not fully recapitulate the hormonal and genetic heterogeneity of human populations [108]. Furthermore, the correlation between tracer binding and disease pathology, such as tau tangles, warrants validation in human post-mortem tissue, as demonstrated in recent studies using human AD brain homogenates [108].

In summary, a tiered translational strategy progressing from rodent screening to NHP validation, coupled with cross-species metabolic profiling and autoradiography on human brain tissue, is essential to minimize the translational pitfalls posed by interspecies differences.

## 5. Conclusions

### 5.1. Evolution of GSK-3β PET Tracer Development

Over the past two decades, GSK-3β PET tracers have evolved from none to over 20 radiolabelled compounds spanning 11 scaffold classes. Major advances include the first brain-penetrant tracer ([^11^C]SB-216763) [81], the first validated target occupancy ([^11^C]PF-367), the first full kinetic modeling in NHPs ([^11^C]OCM-44 [91,92] and [^18^F]OCM-50) [92,93], and the first demonstration of signal differences in a transgenic overexpression mouse ([^18^F]F-CNPI) [90]. Nevertheless, no GSK-3β tracer has yet entered human clinical trials. The root cause appears to be not insufficient affinity or brain uptake, but rather the lack of definitive target-specificity evidence, particularly data directly comparing tracer binding with autoradiography or immunohistochemistry on the same human brain tissue sections.

### 5.2. A Proposed Validation Framework for Candidate Tracers

To facilitate tracer development, a four-tier validation framework is proposed for new GSK-3β PET tracers. Level 1 (chemistry and primary screening) covers in vitro affinity, selectivity over GSK-3α, lipophilicity, passive permeability, and P-gp efflux ratio; the recommended thresholds are derived from empirical data on successful CNS PET tracers and should be viewed as guidelines rather than absolute cut-offs. Level 2 (rodent in vivo screening) evaluates brain uptake, regional distribution, and response to P-gp inhibition, helping to identify candidates with adequate brain penetration before more resource-intensive experiments. Level 3 (target specificity confirmation) verifies target engagement by comparing tracer uptake in GSK-3β-overexpressing transgenic mice versus wild-type or knockout controls, using a structurally similar but inactive analog as a negative control to confirm signal specificity. Level 4 (NHP full quantification) requires full quantitative PET imaging in NHPs, including arterial input function, metabolite-corrected plasma curve, multi-dose blocking for target occupancy, free-fraction-corrected Vτ, and assessment of Vτ stability over time. Tracers that meet the Level 4 criteria would be candidates for subsequent clinical trials.

Applying the above four-tier framework to the most extensively characterized tracers yields a clear translational hierarchy. [^18^F]OCM-50 stands as the most practical tracer, having met Level 4 criteria, despite its low f_p_ limiting signal in low-expression regions [92,93]. [^11^C]OCM-44 also satisfies Level 4, but it is hindered by brain-penetrant metabolites and a short half-life [91,92]. [^18^F]F-CNPI remains at Level 3, awaiting resolution of its P-gp liability [90], while [^11^C]PF-367 is constrained by rapid metabolism and short half-life [22]. Thus, the overall ranking is: [^18^F]OCM-50 > [^11^C]OCM-44 > [^18^F]F-CNPI > [^11^C]PF-367.

### 5.3. Future Innovation Directions

#### 5.3.1. Covalent Inhibitors as PET Tracers

All reported GSK-3β tracers to date are reversible ATP-competitive inhibitors. Covalent inhibitors have recently shown promise as PET probes for other targets, such as KRAS G12C [118], EGFR [119], and ST14 protease [120], suggesting that this strategy could be extended to GSK-3β. For GSK-3β, Cys199 has been identified as a potential binding site for covalent modification by electrophilic warheads, and this approach has been validated across multiple chemotypes, including halomethylketones [37], benzimidazole-based ureas [121], and 1,2,4-thiadiazole-3,5-dione derivatives [122]. Covalent binding offers the potential for higher specificity and sustained target engagement. Future efforts could explore ^18^F-labeled covalent inhibitors designed for irreversible binding, with careful attention to kinetic parameters that influence imaging performance.

#### 5.3.2. Allosteric and Conformation-Selective Probes

GSK-3β activity is regulated by phosphorylation at Ser9 (inhibitory) and Tyr216 (activating). All current ATP-competitive tracers cannot distinguish between these conformational states, as [^3^H]PF-367 binds GSK-3β irrespective of its activation state [91]. One strategy is to develop tracers that recognize phosphorylation-specific epitopes via antibody fragments, affibodies, or conformationally sensitive small molecules. Another is to target allosteric sites, which are less conserved and offer higher selectivity, as validated in multiple CNS targets including mGluR1 ([^18^F]FITM) [123,124], M_4_ ([^11^C]MK-6884, already used in AD patients) [125,126], and EAAT2 ([^18^F]SF-2) [127]. For GSK-3β, known allosteric modulators including TDZD [128], L803-mms [129], and VP0.7 [130] provide chemical starting points. Allosteric probes can report on conformational states, as shown by [^11^C]MK-6884, whose binding is modulated by orthosteric agonists [126]. Despite challenges such as small flexible binding sites and induced-fit mechanisms, probes capable of distinguishing GSK-3β conformations would provide functional information beyond ATP-competitive tracers, making this a promising direction for next-generation PET imaging.

#### 5.3.3. Dual-Modality Probes and Theranostics

In preclinical models, PET/near-infrared fluorescence (NIRF) dual-modality probes have been developed for HER2 [131,132] and ENT1 [133], demonstrating the feasibility of simultaneous whole-body PET imaging and intraoperative fluorescence-guided navigation. This strategy could accelerate translation from rodents to NHPs. Furthermore, because GSK-3β hyperactivation is implicated in multiple diseases, a highly selective tracer could share the same scaffold as a therapeutic inhibitor, forming a theranostic pair that enables patient selection and treatment monitoring in a closed loop.

#### 5.3.4. Validation in Human Brain Tissue

To date, no GSK-3β tracer has been directly compared with phosphorylated tau or GSK-3β immunohistochemistry on human brain tissue sections. Such validation is critical because animal models do not fully recapitulate the regional distribution and post-translational modification status of GSK-3β in the human brain. Before an IND application, it would be valuable to perform autoradiography on post-mortem brain tissue from AD patients and age-matched controls, including hippocampus, cortex, and cerebellum. The signal should be blockable by excess non-radioactive inhibitor and should correlate regionally with GSK-3β expression density. This would provide the strongest available evidence for target specificity in the human context.

#### 5.3.5. Computational Tools for Tracer Design

Beyond traditional medicinal chemistry, computational tools, including molecular docking, molecular dynamics simulations, Artificial Intelligence (AI)—assisted ligand design, and machine learning-assisted property prediction, offer complementary approaches to accelerate GSK-3β PET tracer optimization [134,135,136,137,138]. These methods can help predict blood–brain barrier penetration, P-gp recognition, and metabolic liabilities prior to synthesis, thereby reducing the number of compounds requiring experimental evaluation. However, they cannot replace experimental validation, including target engagement, metabolic profiling, and in vivo assessment, and should be regarded as tools to support design and data analysis, rather than definitive predictive methods.

#### 5.3.6. Integrating Targeted Protein Degradation with PET Imaging

All reported GSK-3β PET tracers are occupancy-based probes that report target expression levels. An alternative approach is to combine PET imaging with targeted protein degradation for non-invasive visualization of target degradation dynamics. This strategy has been validated in PD-L1 (^68^Ga-PET with degradation) [139,140] and Tau (QC-01-175, derived from PET tracer ^18^F-T807) [141]. GSK-3β degraders already exist, including PT-65 (DC_50_ = 34.20 nM in SH-SY5Y cells) [142], brain-penetrant KH1 (DC_50_ in the region of 0.10–1.00 nM in mouse embryonic fibroblasts) [143], and dual GSK3β/CDK5 degraders (DC_50_ = 42 nM in PANC-1 cells) [144]. Brain delivery can be addressed via PROTAC–antibody conjugates or carrier-mediated transport [145]. The convergence of PROTAC technology, PET imaging, and brain delivery offers a promising avenue for next-generation GSK-3β probes capable of simultaneous visualization and modulation of target proteins in the living brain.

In conclusion, the development of GSK-3β PET tracers has progressed significantly over the past two decades, with multiple scaffolds validated in preclinical models and NHP studies. Nevertheless, no tracer has yet demonstrated definitive target-specificity evidence in human brain tissue, and key challenges, including P-gp efflux, brain-penetrant radiometabolites, and low plasma free fraction, remain to be addressed. Future efforts, guided by a more rigorous validation framework and innovative chemical strategies, should prioritize validation in human brain tissue, optimization of pharmacokinetic properties, and exploration of emerging approaches including covalent probes, allosteric and conformation-selective ligands, dual-modality theranostic platforms, and protein degradation-based strategies, among other factors. Continued progress in these directions holds promise for ultimately enabling the clinical translation of GSK-3β PET tracers in Alzheimer’s disease and other tauopathies, thereby providing a powerful tool for early diagnosis and targeted therapy monitoring.

## Figures and Tables

**Table 1:** GSK-3β-targeted PET radiotracers.

Radiotracers	Chemical Scaffolds	Animal Models	Affinity	Brain Uptake	Refs
[^11^C]AR-A014418/ [^11^C-carbonyl]AR-A014418	Urea	SD rat	*K_i_* = 770 nM (AR-A014418)	0.08%ID/g	2005 [78] 2012 [79]
[^11^C]PyrATP-1	Pyrazine	SD rat; Rhesus monkey	*K_i_* = 4.90 nM (PyrATP-1)	SUV ≈ 0.65 after cyclosporine A pretreatment	2014 [80]
[^11^C]SB-216763	Maleimide	Wistar rat; Rhesus monkey	*K_i_* = 9 nM; IC_50_ = 34 nM (SB-216763)	SUV = 2.50 (Rat); SUV = 1.90(NHPs)	2015 [81]
[^18^F]10a		SD rat	IC_50_ = 1.70 nM (10a)	> 0.4%ID/cc	2017 [82]
[^11^C]2		Swiss mouse; 3×Tg-AD mouse	IC_50_ = 890 nM (2)	2.93%ID/g (Swiss)	2022 [83]
[^11^C]Oxadiazole-1-3	Oxadiazole (thioether /sulfoxide/sulfone)	ddY mouse	IC_50_ = 66 nM; 35 nM; 42 nM (Oxadiazole-1–3)	SUV = 1.50; 0.80; 1.60	2015 [84]
[^11^C]CMP	Isonicotinamide	SD rat	IC_50_ = 3.40 nM (CMP)	Minimal uptake (3-fold increase with cyclosporine A)	2019 [85]
[^18^F]10a-d		SD rat	IC_50_ = 1.30 nM; 1.40 nM; 5.80 nM; 3.30 nM (10a-d)	Optimal regional heterogeneity	2021 [86]
[^18^F]F-CNBI [^18^F]F-CNPIFE		FVB/NJ mouse	IC_50_ = 105.30 nM (F-CNBI); IC_50_ = 19.40 nM (F- CNPIFE)	SUV = 0.08 ([^18^F]F-CNBI); SUV = 0.76 ([^18^F]F-CNPIFE)	2023 [87]
[^18^F]28		SD rat;	IC_50_ = 5.20 nM (28)	SUV ≈ 0.51 (homogeneous distribution)	2024 [88]
[^18^F]2 [^18^F]6		FVB/NJ mouse	IC_50_ = 24.29 nM (2); 43.99 nM (6)	SUV = 0.92 ([^18^F]2, 5 min); discontinued in vitro ([^18^F]6)	2024 [88,89]
[^18^F]F-CNPI		Thy1 transgenic mouse	Comparable to 2 and 28	Uptake modulated by P-gp inhibitor	2025 [90]
[^11^C]PF-367	Oxazole-4- carboxamide	Rhesus macaques	IC_50_ = 2.10 nM (PF-367)	SUV = 1.00	2016 [22]
[^11^C]OCM-44		Rhesus macaque	IC_50_ < 2.50 nM (OCM-44)	SUV = 2.00–3.00	2019 [91] 2023 [92]
[^18^F]OCM-49		SD rat	*K_i_* = 0.36 nM (OCM-49)	SUV = 1.50	2021 [93]
[^18^F]OCM-50		SD rat; Rhesus monkey	*K_i_* = 0.28 nM; IC_50_ = 0.80 nM (OCM-50)	SUV ≈ 2.00 (Rhesus monkey)	2021 [93] 2023 [92]
[^11^C]Tideglusib	Thiadiazolidinedione	SD rat	*K_i_* = 60 nM (Tideglusib)	SUV ≈ 0.70	2016 [94]
[^11^C]A1070722	Quinoline-urea	African green monkey	*K_i_* = 0.60 nM (A1070722)	Heterogeneous distribution	2017 [95]
[^18^F]8	Thiazolylacylaminopyridine(TAAP)	KM mouse	IC_50_ = 6.00 nM (8)	1.68%ID/g (2 min)	2023 [96]
[^11^C]13a-e	Imidazolyl pyrimidine	Cynomolgus monkeys	*K_i_* = 4.95 nM; 3.78 nM; 4.39 nM; 4.85 nM; 2.49 nM (13a-e)	Low uptake and rapid clearance	2025 [97]
[^125^I]5/6	Benzofuran-indolylmaleimide	ddY mouse	IC_50_ = 439.90 nM (5); 136.70 nM (6)	1.79/1.76%ID/g SPECT imaging	2016 [98]

”≈” indicates approximate values; semicolons separate multiple values within a single entry. Abbreviations: SD, Sprague–Dawley; NHPs, nonhuman primates; KM, Kunming; SUV, standardized uptake value; %ID/g, percentage of injected dose per gram tissue; %ID/cc, percentage of injected dose per cubic centimeter tissue; P-gp, P-glycoprotein. Standard deviations, where reported in the original literature, are omitted for table clarity and consistency; only mean values are shown.

**Table 2:** Comparison of representative GSK-3β PET tracers with respect to key preclinical and translational parameters.

Parameter	[^11^C]PF-367/[^11^C]OCM-44	[^18^F]OCM-50	[^18^F]F-CNPI
Affinity (GSK-3β)	IC_50_ = 2.10 nM (PF-367); IC_50_ < 2.50 nM (OCM-44)	*K_i_* = 0.28 nM (OCM-50)	nM range, from prior study (F-CNPI)
Isoform selectivity	Moderate (dual α/β, PF-367); OCM-44: high	High (both isoforms)	Not established
Brain uptake (NHP/mouse)	NHP: SUV = 1.00 ([^11^C]PF-367); Vτ = 1.40–2.90 mL/cm^3^ ([^11^C]OCM-44)	Rodent SUV ~2.00; NHP Vτ = 0.39–0.87 mL/cm^3^	SUV = 0.92 in transgenic mice (requires P-gp inhibition)
Metabolite issues	Brain-penetrant metabolites for OCM-44	No brain-penetrant metabolites; no defluorination	No reported
Blocking validation	>80% target occupancy (Vτ/f_p_-corrected)	>80% target occupancy (Vτ/f_p_-corrected)	Not fully confirmed
^18^F-labeling feasible	No (^11^C only)	Yes	Yes
Overexpression model validation	No	No	Yes (Thy1-GSK-3β mouse)
Current translational stage	Preclinically well-validated, but short ^11^C half-life	Full NHP quantification completed; *f_p_* issue remains	Mechanistic doubts; P-gp liability

## Data Availability

No new data were created or analyzed in this study. Data sharing is not applicable to this article.

## Abbreviations

The following abbreviations are used in this manuscript:

%ID/g	Percentage of injected dose per gram tissue
3×Tg-AD	Triple Transgenic Alzheimer’s Disease (mouse model)
AD	Alzheimer’s disease
ADME	Absorption, distribution, metabolism, and excretion
AGD	Argyrophilic grain disease
Akt	Protein Kinase B (PKB)
AI	Artificial intelligence
APP	Amyloid precursor protein
ATP	Adenosine triphosphate
AUC	Area under the curve
Aβ	Amyloid-β
BBB	Blood–brain barrier
BD	Bipolar disorder
BEMP	2-tert-Butylimino-2-diethylamino-1,3-dimethylperhydro-1,3,2-diazaphosphorine
Bmax	Maximum binding capacity
BPND	Binding potential (non-displaceable)
CBD	Corticobasal degeneration
CH2Cl2	Dichloromethane
CNS	Central nervous system
DC50	Half maximal degradation concentration
DMAc	N,N-Dimethylacetamide
DMAP	4-Dimethylaminopyridine
DIPEA	N,N-Diisopropylethylamine
EDCI	1-Ethyl-3-(3-dimethylaminopropyl)carbodiimide
EGFR	Epidermal growth factor receptor
ENT1	Equilibrative nucleoside transporter 1
Et3N	Triethylamine
GSK-3β	Glycogen synthase kinase-3β
HATU	O-(7-azabenzotriazol-1-yl)-N,N,N′,N′-tetramethyluronium hexafluorophosphate
HER2	Human epidermal growth factor receptor 2
HD	Huntington’s disease
HLM	Human liver microsomes
HTS	High-throughput screening
HTT	Huntingtin
IC50	Half maximal inhibitory concentration
IND	Investigational New Drug (application)
K222	4,7,13,16,21,24-Hexaoxa-1,10-diazabicyclo [8.8.8]hexacosane
KRAS-G12C	Kirsten rat sarcoma viral oncogene homolog (KRAS) G12C mutation
Kd	Dissociation constant
Ki	Inhibition constant
LogD7.4	Distribution coefficient at pH 7.4
LogP	Partition coefficient
LPS	Lipopolysaccharide
m-CPBA	meta-Chloroperoxybenzoic Acid
MDCK	Madin–Darby canine kidney
MDR1	Multidrug Resistance Protein 1
MST	Microscale thermophoresis
NFT	Neurofibrillary tangle
NHP	Nonhuman primate
NIRF	Near-infrared fluorescence
NRB	Number of rotatable bonds
P-gp	P-glycoprotein
PAMPA	Parallel artificial membrane permeability assay
PD	Parkinson’s disease
PET	Positron emission tomography
PiD	Pick’s disease
PI3K	Phosphoinositide 3-Kinase
pSer9	Phosphorylated Serine 9
PSP	Progressive supranuclear palsy
pY216	Phosphorylated Tyrosine 216
QC	Glutaminyl cyclase
RCY	Radiochemical yield
r.t.	room temperature
SAR	Structure–activity relationship
SD	Sprague–Dawley
SPECT	Single-photon emission computed tomography
ST14	Suppression of tumorigenicity 14
SUV	Standardized uptake value
TAAP	Thiazolylacylaminopyridine
TBAF	Tetrabutylammonium Fluoride
TBAOH	Tetrabutylammonium Hydroxide
TBSCl	tert-Butyldimethylsilyl Chloride
TEAF	Tetraethylammonium Fluoride
tPSA	Topological polar surface area
TPTU	O-[2-oxo-1(2H)-pyridyl]-N,N,N′,N′-tetramethyluronium tetrafluoroborate
Vτ	Total distribution volume
Xantphos	4,5-Bis(diphenylphosphino)-9,9-dimethylxanthene
